# Phenotypic Characterization of Transgenic Mice Expressing Human IGFBP-5

**DOI:** 10.3390/ijms22010335

**Published:** 2020-12-30

**Authors:** Xinh-Xinh Nguyen, Matthew Sanderson, Kristi Helke, Carol Feghali-Bostwick

**Affiliations:** 1 Division of Rheumatology & Immunology, Department of Medicine, Medical University of South Carolina, Charleston, SC 29425, USA; nguyenxi@musc.edu (X.-X.N.); sandemat@musc.edu (M.S.); 2 Department of Comparative Medicine, Medical University of South Carolina, Charleston, SC 29425, USA; helke@musc.edu; 3 Department of Pathology and Laboratory Medicine, Medical University of South Carolina, Charleston, SC 29425, USA

**Keywords:** insulin-like growth factor binding protein-5 (IGFBP-5), fibrosis, extracellular matrix (ECM), transgenic model

## Abstract

Pulmonary fibrosis is one of the important causes of morbidity and mortality in fibroproliferative disorders such as systemic sclerosis (SSc) and idiopathic pulmonary fibrosis (IPF). Insulin-like growth factor binding protein-5 (IGFBP-5) is a conserved member of the IGFBP family of proteins that is overexpressed in SSc and IPF lung tissues. In this study, we investigated the functional role of IGFBP-5 in the development of fibrosis in vivo using a transgenic model. We generated transgenic mice ubiquitously expressing human IGFBP-5 using CRISPR/Cas9 knock-in. Our data show that the heterozygous and homozygous mice are viable and express human IGFBP-5 (h*IGFBP-5*). Transgenic mice had increased expression of extracellular matrix (ECM) genes, especially *Col3a1*, *Fn,* and *Lox* in lung and skin tissues of mice expressing higher transgene levels. Histologic analysis of the skin tissues showed increased dermal thickness, and the lung histology showed subtle changes in the heterozygous and homozygous mice as compared with the wild-type mice. These changes were more pronounced in animals expressing higher levels of h*IGFBP-5*. Bleomycin increased ECM gene expression in wild-type mice and accentuated an increase in ECM gene expression in transgenic mice, suggesting that transgene expression exacerbated bleomycin-induced pulmonary fibrosis. Primary lung fibroblasts cultured from lung tissues of homozygous transgenic mice showed significant increases in ECM gene expression and protein levels, further supporting the observation that IGFBP-5 resulted in a fibrotic phenotype in fibroblasts. In summary, transgenic mice expressing human IGFBP-5 could serve as a useful animal model for examining the function of IGFBP-5 in vivo.

## 1. Introduction

Pulmonary fibrosis is a complication of several different diseases such as systemic sclerosis (SSc, scleroderma) and idiopathic pulmonary fibrosis (IPF). SSc is a complex autoimmune disease characterized by progressive fibrosis of the skin and multiple visceral organs [1,2]. Despite active research, the etiology of this connective tissue disease, which causes high morbidity and mortality, is still unknown. Lung fibrosis is also the hallmark of IPF. In fact, IPF and SSc, while being different diseases, show some similarities [3,4]. Pulmonary fibrosis in both of these diseases is characterized by the overproduction of extracellular matrix (ECM) components such as collagen and fibronectin in the lung. To date, the therapeutic options for patients with pulmonary fibrosis are limited, and lung transplantation remains the only viable treatment [2]. Therefore, identifying novel therapeutic targets and establishing a translatable study model would significantly advance the treatment and understanding of SSc-associated lung disease and related diseases such as IPF.

Insulin-like growth factor binding-5 (IGFBP-5) is the most conserved member of the IGFBP family and binds IGF-1 and IGF-2 with high affinity [5,6]. Although IGFBPs can regulate IGF activity, they also have IGF-independent effects [5]. Similar to other IGFBPs, IGFBP-5 also exerts both IGF-dependent and -independent effects [5,7,8]. We previously demonstrated that IGFBP-5 was overexpressed in dermal fibroblasts from patients with SSc and pulmonary fibroblasts from patients with IPF and triggered a fibrotic phenotype in vitro in primary human pulmonary fibroblasts [7,8]. We recently reported that IGFBP-5 levels were significantly increased in lung tissues and primary pulmonary fibroblasts of patients with SSc-associated pulmonary fibrosis [9]. In addition, IGFBP-5 induced fibrosis in vivo in mouse skin and lung tissues [10,11], and ex vivo in human skin maintained in organ culture [5,12]. Taken together, our findings suggest that IGFBP-5 mediates fibrosis in more than one organ and at least two diseases (SSc and IPF).

We previously established that recombinant and adenovirally expressed IGFBP-5 induced a fibrotic phenotype in vitro in primary human pulmonary fibroblasts [7,8], in vivo in mouse skin and lung tissues [10,11], and ex vivo in human skin and lung tissues maintained in organ culture [5,11,13], respectively. However, our previous models relied on the use of adenovirally expressed *IGFBP-5*. Therefore, the purpose of this study was to investigate the functional role of IGFBP-5 in the development of fibrosis in vivo using a transgenic model. To do so, we generated transgenic mice ubiquitously expressing human *IGFBP-5* using CRISPR/Cas9 knock-in. Our data show that the heterozygous and homozygous mice are viable and express the transgene. Thus, in this study, our goal was to characterize the phenotype of transgenic mice expressing human *IGFBP-5* and to determine the functional role of IGFBP-5 in the development of fibrosis in vivo.

## 2. Results

### 2.1. Generation of Transgenic IGFBP-5 Mice

**Figure 1:**
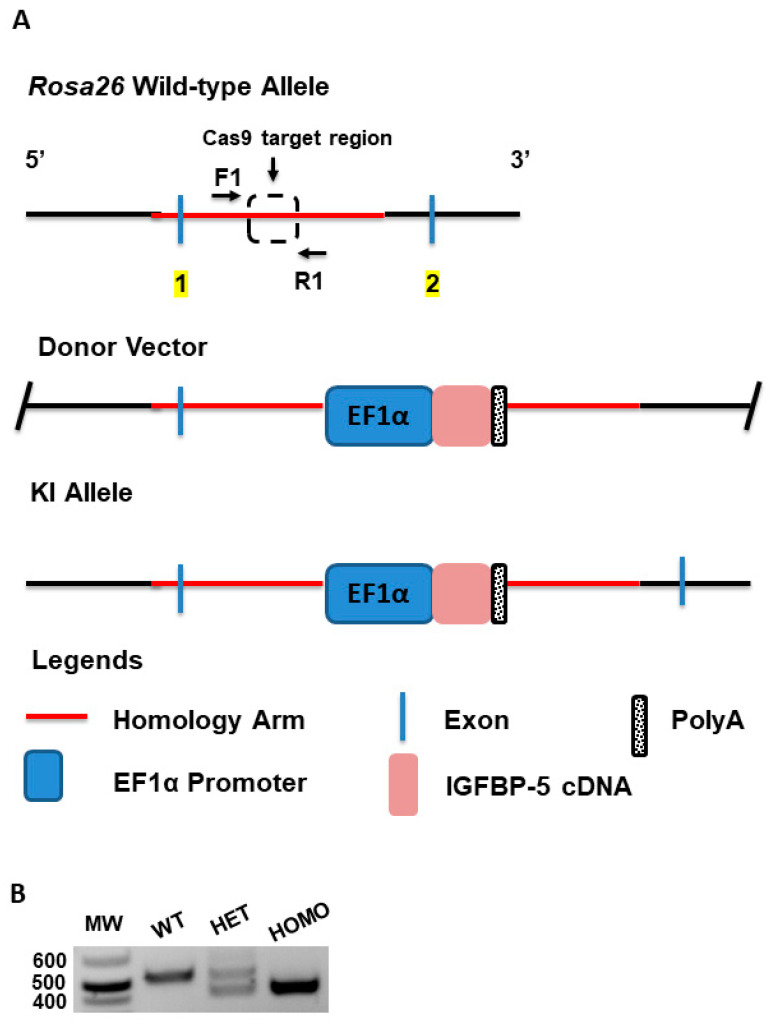
Generation and characterization of IGFBP-5 mice. (**A**) Schematic of generation of IGFBP-5 transgenic mice; (**B**) Genotype of mice detected on agarose gel. The key information is noted as follows: MW, DNA ladder; WT, wild-type; HET, heterozygous; HOMO, homozygous.

A schematic describing the generation of the IGFBP-5 transgenic mice is shown in Figure 1A. The elongation factor 1 α (EF1α) promoter was used to drive expression of the transgene since the EF1α promoter is a powerful mammalian promoter [13]. The *Rosa26* locus on chromosome 6 of the mouse genome was used as a target site because it is frequently used for integration of transgene constructs to achieve ubiquitous gene expression in mice [14]. Mice were weaned at the age of 21 days and tail samples were collected for genotyping. As seen in Figure 1B and Supplemental Figure S1, a single band at 607 bp is indicative of wild-type mice. Heterozygous mice are indicated with two bands at 607 bp (upper band) and 480 bp (lower band), and homozygous mice are indicated with a single band at 480 bp. In addition, the body weight of both male and female mice was measured at eight-weeks of age. As shown in Supplemental Figure S2A,B, heterozygous and homozygous mice of both sexes had slightly lower body weights as compared with the wild-type littermate mice. It is noted that the mice were given the same amount of food, water, and quality of care. Therefore, the differences in body weights between the wild-type and transgenic mice were probably not due to differences in food consumption, but may have resulted from a change in energy homeostasis, as reported in other models [15]. We also looked at whole body images of both male and female transgenic mice. There were no apparent differences among the genotypes (Supplemental Figure S2C). The femur and tibia length were also measured and were comparable among the groups. (Supplemental Figure S2D,E).

### 2.2. Expression of Human IGFBP-5

**Figure 2:**
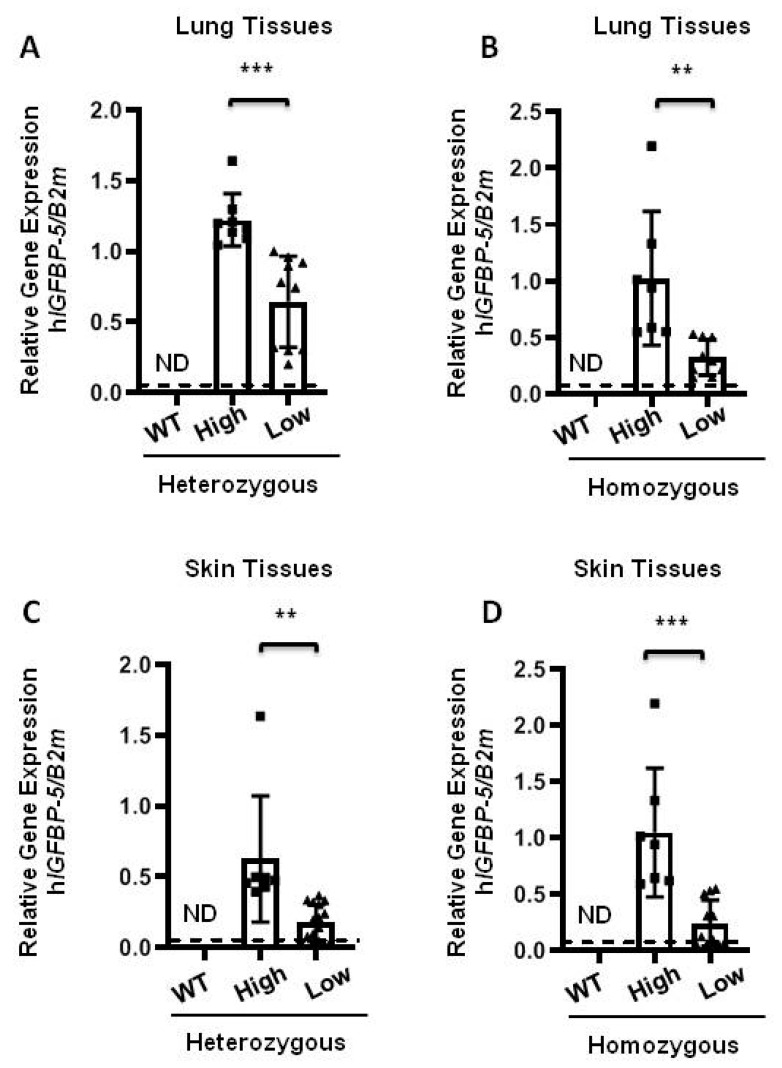
Increased gene expression of human *IGFBP-5* in transgenic mice. Lung and skin tissues of transgenic mice were harvested. (**A**,**B**) Relative gene expression of human *IGFBP-5* in lung tissues of heterozygous and homozygous mice expressing high (square) and low (triangle) levels of transgene was measured by qPCR. Data were obtained from 18 HET (8 high and 10 low) and 16 HOMO (7 high and 9 low) mice; (**C**,**D**) Relative gene expression of human *IGFBP-5* in skin tissues of heterozygous and homozygous mice expressing high and low levels of the transgene was measured by qPCR. Data were obtained from 18 HET (8 high and 10 low) and 16 HOMO (7 high and 9 low) mice. The dotted lines indicate undetected values in the WT group (ND, non-detectable). Data was analyzed using unpaired *t*-test for comparison between h*IGFBP-5* levels in the high and low expressing mice. Values represent mean ± standard deviation. ** *p* < 0.01 and *** *p* < 0.001.

In order to examine the expression of the human *IGFBP-5* (h*IGFBP-5*) transgene, lung and skin tissues of eight-week-old mice were harvested for measurement of baseline levels of transgene and ECM gene expression. To characterize the expression of the h*IGFBP-5* transgene, the transgene copy number was calculated in both heterozygous and homozygous mice using a primer amplifying the h*IGFBP-5* sequence. High and low copy numbers of the transgene from heterozygous and homozygous mice were detected. As seen in Figure 2A,B, both heterozygous and homozygous mice express the human transgene, in contrast to wild-type mice. Mice with higher transgene expression levels are shown as high expressor (high) as compared with low expressor (low) mice. As expected, the level of h*IGFBP-5* in the wild-type group was undetectable. A similar observation was made in skin tissues of heterozygous and homozygous transgenic mice (Figure 2C,D).

### 2.3. The Effect of Transgenic hIGFBP-5 on Extracellular Matrix Gene Expression in Lung

**Figure 3:**
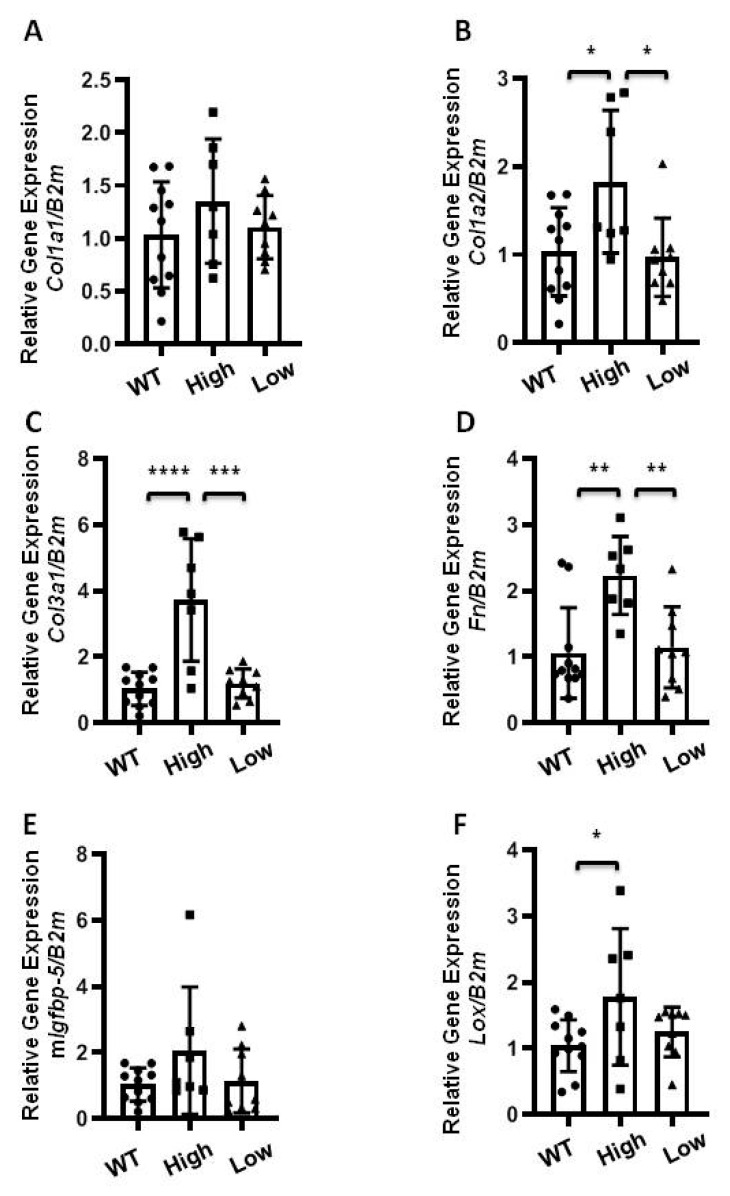
Extracellular matrix (ECM) gene expression in lung tissues of homozygous mice expressing higher levels of transgene. Lung tissues of homozygous mice expressing high (square) and low (triangle) levels of h*IGFBP-5* at 8 weeks of age were harvested to measure baseline expression using qPCR. The following genes were analyzed: (**A**) *Col1a1*; (**B**) *Col1a2*; (**C**) *Col3a1*; (**D**) *Fn*; (**E**) Mouse *Igfbp-5*; (**F**) *Lox*. Data were obtained from 11 WT (6 M and 5 F) (circle), 7 homozygous high h*IGFBP-5* expressing (5 M and 2 F) and 9 homozygous low h*IGFBP-5* expressing (6 M and 3 F) mice. A statistical comparison was performed using one-way ANOVA followed by Tukey’s multiple comparisons test. Values represent mean ± standard deviation. * *p* < 0.05, ** *p* < 0.01, *** *p* < 0.001, and **** *p* < 0.0001.

The lung tissues of eight-week-old mice were harvested to examine baseline expression of ECM genes. Comparative analysis of the levels of h*IGFBP-5* expression were done in heterozygous and homozygous mice expressing high and low levels of the transgene. As seen in Figure 3A, gene expression levels of *Col1a1* were measured in homozygous mice expressing high and low levels of h*IGFBP-5*. There was no significant induction of *Col1a1* levels among the groups, although there was a trend for increased *Col1a1* in the high expressors. However, levels of *Col1a2*, *Col3a1*, and *Fn* were significantly increased in mice expressing high levels of h*IGFBP-5* as compared with wild-type mice and mice with low expression of h*IGFBP-5* (Figure 3B–D). The gene expression levels of endogenous mouse *Igfbp-5* were also examined. As seen in Figure 3E, there was no difference in m*Igfbp-5* levels among the groups. Expression levels of the ECM crosslinking enzyme lysyl oxidase (*Lox*) were also quantified. *Lox* levels were significantly increased in mice expressing high levels of h*IGFBP-5* as compared with wild-type mice (Figure 3F). In addition, lung tissues from homozygous mice expressing high and low levels of h*IGFBP-5* and wild-type mice were used for protein analysis. As seen in Supplemental Figure S3A,B, the protein levels of Col1α1 were significantly increased in mice expressing higher levels of h*IGFBP-5* as compared with wild-type mice.

The effects of h*IGFBP-5* levels were also examined in heterozygous mice expressing high and low levels of h*IGFBP-5* in lung tissues. As seen in Supplemental Figure S4, only gene expression levels of *Col1a1* and *Fn* were significantly increased in heterozygous mice expressing high levels of h*IGFBP-5* (Supplemental Figure S4A,D), whereas low transgene-expressing mice had significantly higher *Col1a2* and *Col3a1* levels (Supplemental Figure S4B,C) and comparable levels of m*Igfbp-5* and *Lox* (Supplemental Figure S4E,F). Since homozygous mice expressing higher transgene levels showed increased ECM production, additional analyses were done using these mice.

### 2.4. The Effect of Transgenic hIGFBP-5 on Extracellular Matrix Gene Expression in Skin

**Figure 4:**
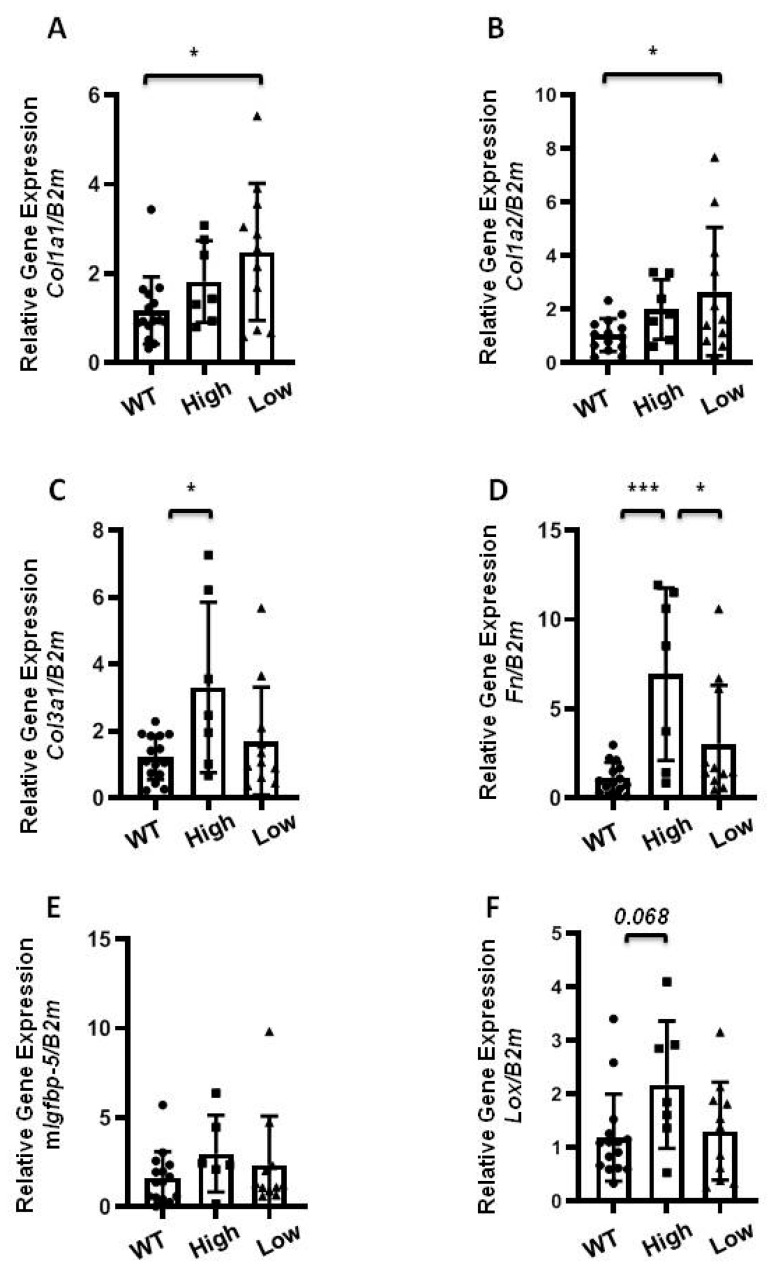
ECM gene expression in skin tissues of homozygous mice expressing higher levels of h*IGFBP-5* at 8 weeks of age were harvested to measure baseline expression using qPCR. The following genes were analyzed: (**A**) *Col1a1*; (**B**) *Col1a2*; (**C**) *Col3a1*; (**D**) *Fn*; (**E**) Mouse *Igfbp-5*; (**F**) *Lox*. Data were obtained from 15 WT (8 M and 7 F) (circle), 7 homozygous high h*IGFBP-5* expressing (7 M) (square), and 11 homozygous low h*IGFBP-5* expressing (5 M and 6 F) (triangle) mice. A statistical comparison was performed using one-way ANOVA followed by Tukey’s multiple comparisons test. Values represent mean ± standard deviation. * *p* < 0.05 and *** *p* < 0.001.

To further examine the effect of the transgene, we examined skin as it is another organ affected by fibrosis in SSc patients. Mice expressing high and low h*IGFBP-5* levels demonstrated increased expression of *Col1a1* and *Col1a2* that reached statistical significance in mice expressing low h*IGFBP-5* levels (Figure 4A,B). The gene expression levels of *Col3a1* and *Fn* were significantly upregulated in mice expressing high levels of h*IGFBP-5* as compared with low expressor mice (Figure 4C,D), comparable to levels observed in lung tissues. Mice expressing low h*IGFBP-5* levels produced comparable levels of *Col3a1* and *Fn* as compared with wild-type mice (Figure 4C,D). The endogenous levels of mouse *Igfbp-5* were comparable in the three groups of mice (Figure 4E). Similar to its expression in lung tissues, levels of *Lox* mRNA were increased in the high h*IGFBP-5* expressor group as compared with the low expressor group, although the difference did not reach statistical significance (*p* = 0.068).

The effects of h*IGFBP-5* levels were also examined in skin tissues of heterozygous mice. The levels of genes of interest (*Col1a1*, *Col1a2*, *Col3a1*, m*Igfbp-5,* and *Lox*) were comparable among the groups (Supplemental Figure S5A–C,E,F). However, *Fn* expression levels were significantly higher in high transgene-expressing mice as compared with low transgene-expressing and wild-type mice (Supplemental Figure S5D).

### 2.5. Histological Evaluation of Lung and Skin Tissues

**Figure 5:**
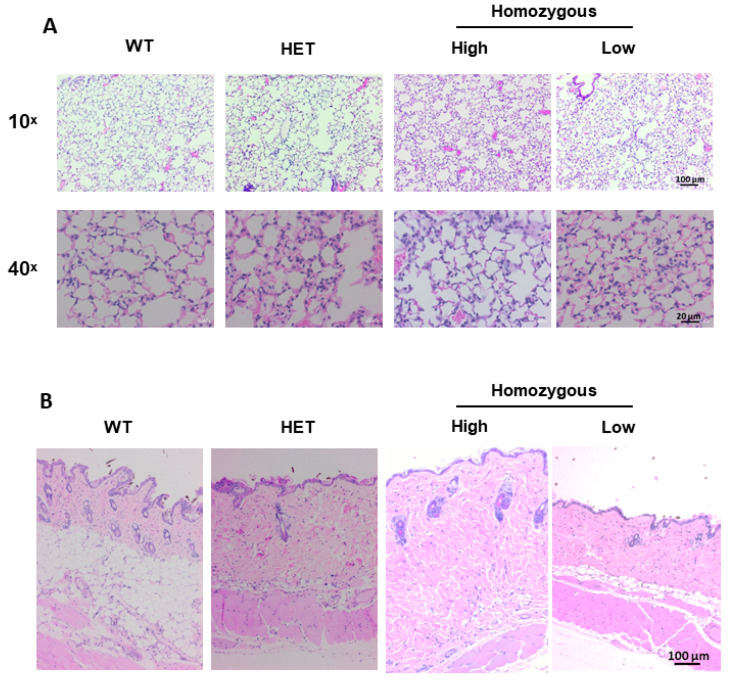
Histological evaluation of tissue morphology in lung and skin tissues. Lung and skin tissue sections of 8-week-old mice were stained with hematoxylin and eosin (H&E). (**A**) Representative images of lung tissues of wild-type, heterozygous, and homozygous mice expressing high and low levels of h*IGFBP-5*. Magnification 10×, scale bar 100 µm, and magnification 40×, scale bar 20 µm; (**B**) Representative images of skin tissues of wild-type, heterozygous, and homozygous mice expressing high and low levels of h*IGFBP-5*. Magnification 10×, scale bar 100 µm.

Histological sections of lung and skin tissues were examined in wild-type, heterozygous and homozygous mice expressing high and low levels of transgene. Histopathologic evaluation was completed on hematoxylin and eosin (H&E) stained (Figure 5) and Masson trichrome-stained (Supplemental Figure S6) lung and skin tissues. There were slight and subtle changes to the lungs of heterozygous and homozygous animals as compared with wild-type mice which were more pronounced in high transgene-expressing mice (Figure 5A and Supplemental Figure S6A). There was minimal accumulation of amorphous pale amphophilic material in few alveoli adjacent to alveolar walls consistent with fibrin or increased extracellular matrix deposition. Increased cellularity with increased numbers of inflammatory cells in alveolar walls and infrequently in the increased inter-alveolar matrix was also noted. The inflammatory cells were primarily macrophages. There were minimally increased numbers of alveolar type II pneumocytes, although a few random fibroblasts in these areas cannot be ruled out. Since inflammatory cells were observed, we also examined the expression of the pro-fibrotic and inflammatory cytokine interleukin-6 (IL-6) whose expression is increased in SSc fibroblasts and SSc patient serum [16,17]. As seen in Supplemental Figure S7A, gene expression levels of *Il-6* showed an increasing trend in lung tissues of mice expressing high levels of transgene, while its levels were comparable in skin tissues (Supplemental Figure S7B).

The dermis of the high transgene-expressing mice showed greater collagen content resulting in a thickened dermis and decreased hypodermal layer regardless of hair cycle (Figure 5B and Supplemental Figure S6B,C). In wild-type mice, the collagen bundles were regularly woven, although not in a distinct pattern. In homozygous mice expressing high levels of h*IGFBP-5*, the collagen bundles were crowded and admixed with increased cells including multinucleated cells and homogenous acellular eosinophilic material consistent with extracellular matrix.

### 2.6. The Effect of Transgene Expression in Aging Mice

**Figure 6:**
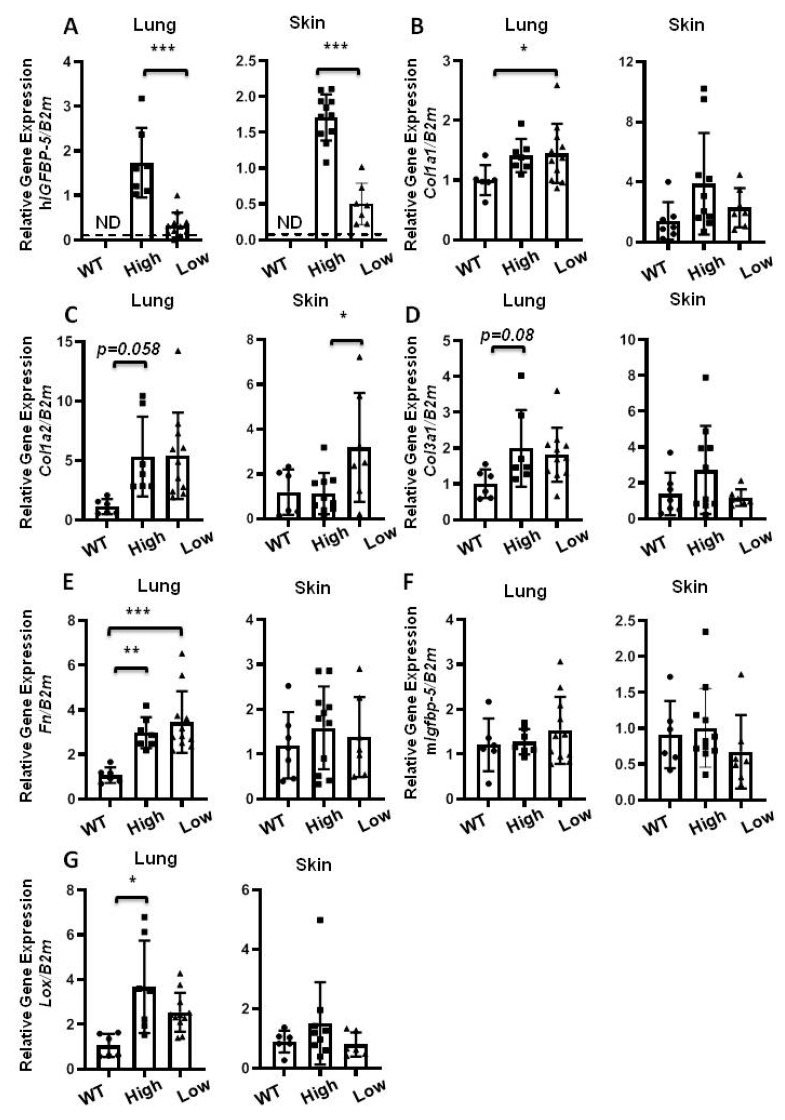
Gene expression of aged mice. Lung and skin tissues of homozygous 1-year old mice expressing high and low levels of the transgene were harvested for qPCR. The following genes were analyzed: (**A**) h*IGFBP-5*; (**B**) *Col1a1*; (**C**) *Col1a2*; (**D**) *Col3a1*; (**E**) *Fn*; (**F**) m*Igfbp-5*; (**G**) *Lox*. Data were obtained from 7 WT (5 M and 2 F) (circle), 11 high h*IGFBP-5* expressing (4 M and 7 F) (square) and 7 low h*IGFBP-5* expressing (3 M and 4 F) (triangle) mice. A statistical comparison was performed using one-way ANOVA followed by Tukey’s multiple comparisons test. Values represent mean ± standard deviation. * *p* < 0.05, ** *p* < 0.01, and *** *p* < 0.001.

Since older mice are more susceptible to lung fibrosis [18], next, we examined the effect of h*IGFBP-5* expression in aging wild-type and homozygous mice expressing high and low levels of the transgene (Figure 6A). Gene expression levels of *Col1a1* increased in lung tissues and showed an increasing trend in skin tissues of transgenic mice as compared with wild-type mice (Figure 6B). Gene expression levels of *Col1a2* and *Col3a1* showed an increasing trend in lung and skin tissues of transgenic mice as compared with wild-type mice (Figure 6C,D). Expression of *Fn* was significantly increased in lung tissues, but not skin tissues, of transgenic mice, irrespective of the level of transgene expression (Figure 6E). Endogenous m*Igfbp-5* expression was comparable across the groups (Figure 6F). *Lox* mRNA levels in lung tissues were significantly increased in high transgene-expressing mice, but showed no significant differences in skin tissues (Figure 6G). Overall, our findings suggest that the response to transgene expression was different in lung and skin tissues of aged mice.

**Figure 7:**
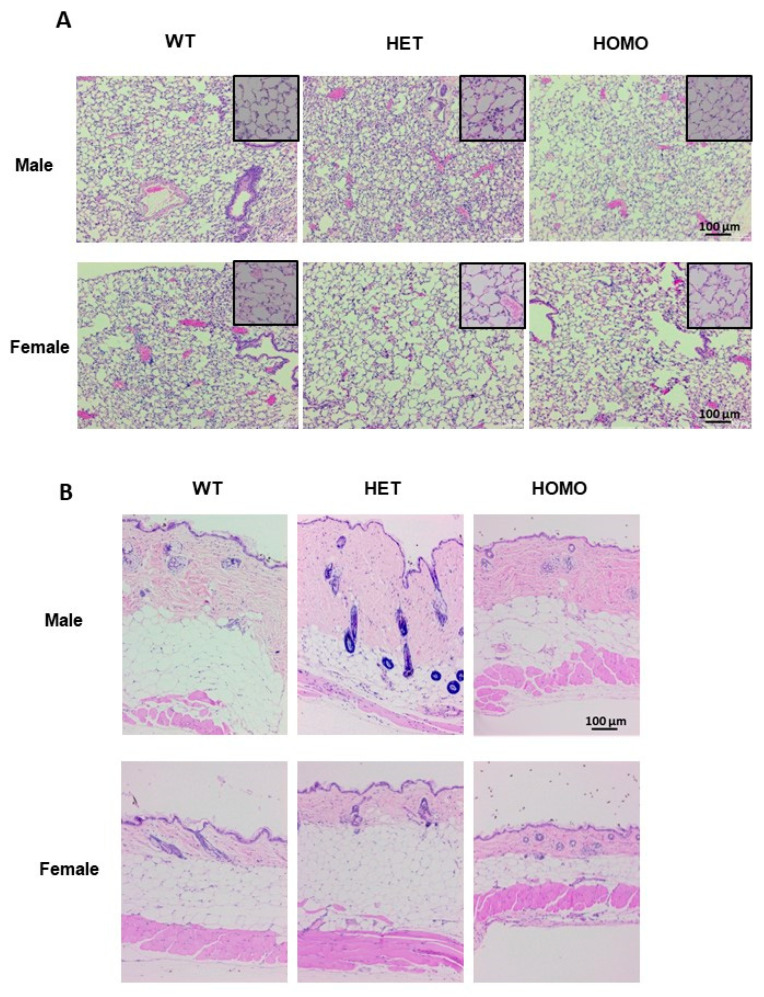
Histological evaluation of tissue morphology in lung and skin from aged mice. Lung and skin tissue sections from 1-year old mice were used for H&E staining. (**A**) Representative images of lung tissues of male and female mice. Magnification 10×, scale bar 100 µm, and inset magnification 40×, scale bar 20 µm; (**B**) Representative images of skin tissues of male and female mice. Magnification 10×, scale bar 100 µm.

In the lung tissues, evidence of collagen deposition was noted in heterozygous male mice (Figure 7A and Supplemental Figure S8A, upper panel), while lung morphology was comparable in female mice irrespective of genotype (Figure 7A and Supplemental Figure S8A, lower panel). The heterozygous male mice displayed an increased dermal thickness as compared with the wild-type mice. Although the dermal thickness of the homozygous males was comparable to that of the wild-type mice, the collagen bundles exhibited a denser arrangement (Figure 7B and Supplemental Figure S8B, upper panel). In general, one-year-old male mice had thicker skin as compared with female mice (Figure 7B). Dermal thickness of the aged female mice was comparable irrespective of genotype (Figure 7B and Supplemental Figure S8B, lower panel). Furthermore, aged mice had an increased hypodermal adipose layer, which was expected in older mice since they increased in weight as they aged.

It is worth noting that no significant histological changes were observed in other tissues. No evidence of fibrosis was noted in kidneys (Supplemental Figure S9), liver (Supplemental Figure S10), or heart (Supplemental Figure S11) of heterozygous or homozygous male or female transgenic mice, irrespective of age.

### 2.7. The Effect Transgene Expression on Bleomycin-Induced Pulmonary Fibrosis

**Figure 8:**
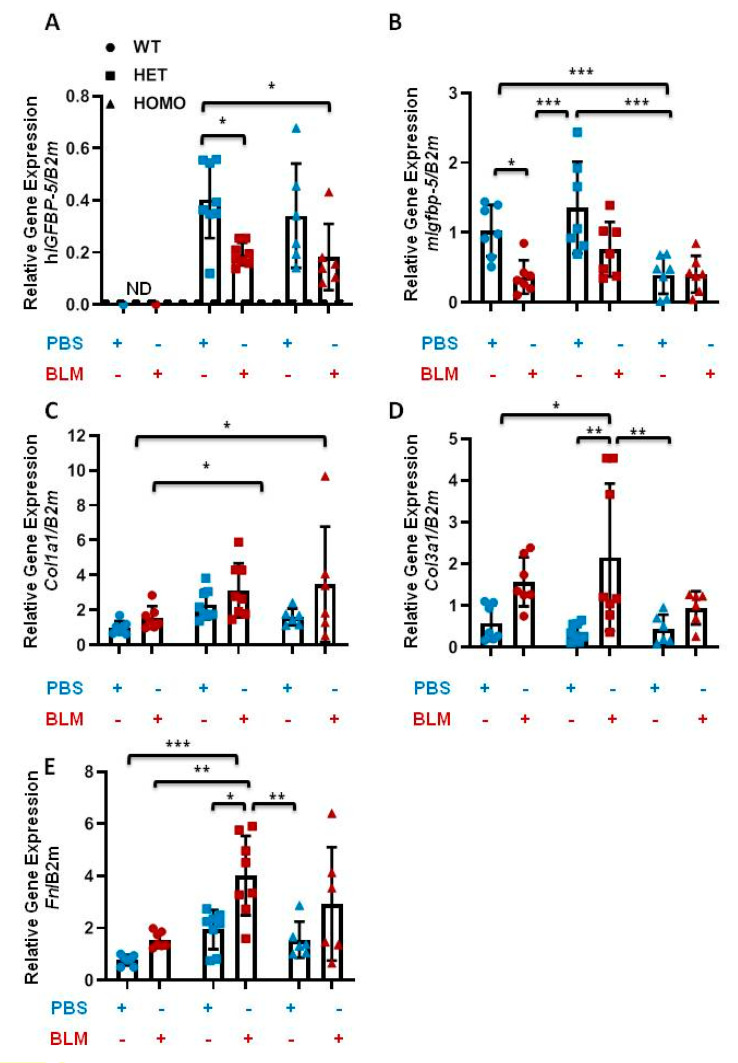
Bleomycin treatment increases ECM gene expression in transgenic mice. Eight-week-old male and female mice were examined 14 days after bleomycin (BLM, dark orange) or vehicle (PBS, blue) administration. The following genes were analyzed in lung tissues: (**A**) h*IGFBP-5*; (**B**) m*Igfbp-5*; (**C**) *Col1a1*; (**D**) *Col3a1*; (**E**) *Fn*. Data were obtained from 7 WT (4 M and 3 F) (circle), 8 HET (4 M and 4 F) (square), and 6 HOMO (3 M and 3 F) (triangle) mice. A statistical comparison was performed using two-way ANOVA followed by Tukey’s multiple comparisons test. Values represent mean ± standard deviation. * *p* < 0.05, ** *p* < 0.01, and *** *p* < 0.001.

Bleomycin (BLM)-induced murine pulmonary fibrosis is the most common experimental model of lung fibrosis [19]. To determine if h*IGFBP-5* transgene expression modulates BLM-induced lung fibrosis, BLM was administered via the oropharyngeal route to eight-week-old male and female mice. Lung tissues were harvested after two weeks. Human *IGFBP-5* was not detected in wild-type mice irrespective of treatment, as expected (Figure 8A). In the presence of BLM, both heterozygous and homozygous mice showed a reduction in h*IGFBP-5* transgene levels (Figure 8A). Similarly, the endogenous levels of mouse *Igfbp-5* were reduced in wild-type, heterozygous, and homozygous mice that received BLM as compared with the groups that received vehicle control (Figure 8B). Furthermore, BLM treatment increased expression levels of the ECM genes *Col1a1*, *Col3a1,* and *Fn,* and these levels showed a trend of further increase in heterozygous and homozygous mice treated with BLM as compared with wild-type mice treated similarly (Figure 8C–E), suggesting that h*IGFBP-5* transgene expression potentiated the response to BLM.

### 2.8. The Effect of hIGFBP-5 Transgene Expression on the Phenotype of Lung Fibroblasts

**Figure 9:**
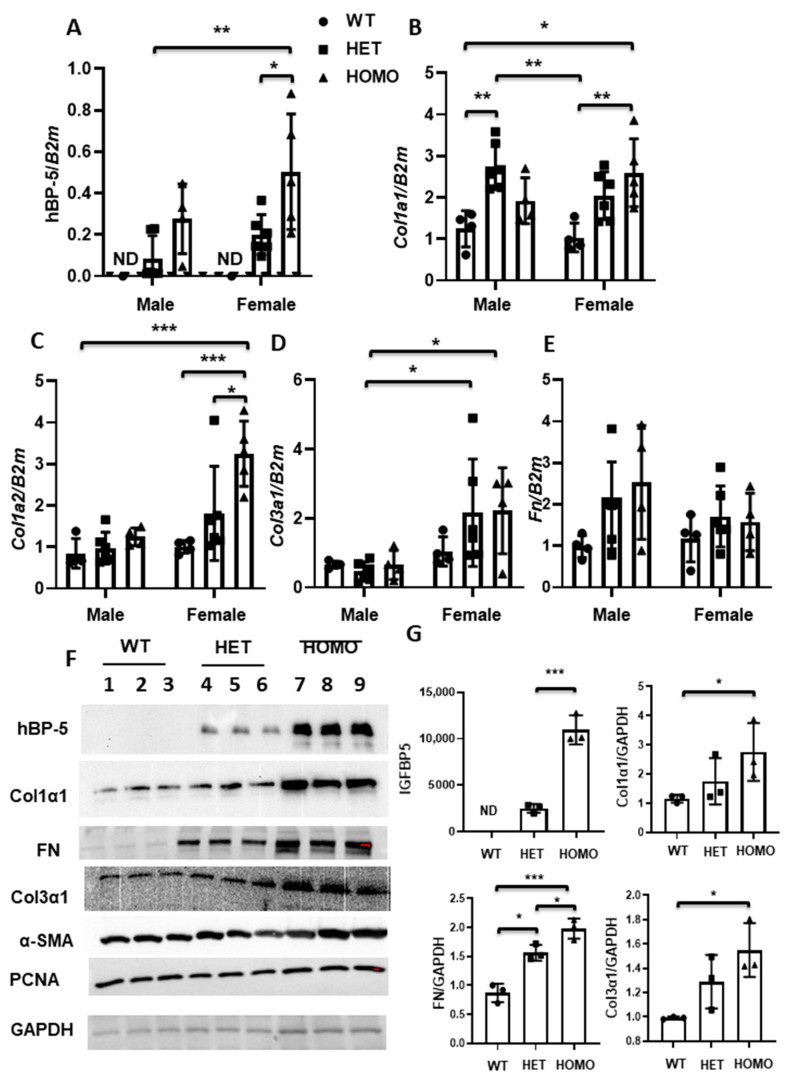
Increased ECM production in cultured lung fibroblasts of *hIGFBP5* transgenic mice. Primary lung fibroblasts were cultured from lung tissues of male (blue) and female (dark orange) non-transgenic and transgenic mice. An equal number of primary lung fibroblasts were plated, and supernatants and RNA samples were harvested for Western Blot and qPCR, respectively. Cells were used in passages 3 to 7. The following genes were analyzed: (**A**) h*IGFBP-5*; (**B**) *Col1a1;* (**C**) *Col1a2*; (**D**) *Col3a1*; (**E**) *Fn*. The data were obtained from primary lung fibroblasts of 4 WT females, 6 HET females, 5 HOMO females, 4 WT males, 6 HET males, and 4 HOMO males. A statistical comparison was performed using two-way ANOVA followed by Tukey’s multiple comparisons test. Values represent mean ± standard deviation. * *p* < 0.05, ** *p* < 0.01, and *** *p* < 0.001; (**F**) Protein levels of hIGFBP-5 (supernatants), collagen alpha (I) (Col1α1), FN, collagen alpha (III) (Col3α1), alpha-SMA, PCNA, and GAPDH (cellular lysates) in fibroblasts cultured from female mice; (**G**) Graphical presentation of data from F, *n* = 3 per group. For hBP-5, ND, non-detectable. Data were analyzed using the unpaired t-test for comparison between the hIGFBP-5 levels in the heterozygous and homozygous group. Values represent mean ± standard deviation. *** *p* < 0.001. For Col1α1, FN, and Col3α1, statistical comparison was performed using one-way ANOVA followed by Tukey’s multiple comparisons test. Values represent mean ± standard deviation. * *p* < 0.05 and *** *p* < 0.001.

We reported that IGFBP-5 levels were increased in dermal fibroblasts of patients with SSc and pulmonary fibroblasts of patients with SSc and IPF [7,9,20]. IGFBP-5 is known to be cell type- and tissue-specific, and since the effects of h*IGFBP-5* were variable in lung and skin tissues in vivo, primary lung fibroblasts from mouse lung tissues were cultured to assess the effects of h*IGFBP-5* in vitro. Expression levels of h*IGFBP-5* were greater in fibroblasts from homozygous mice as compared with heterozygous mice (Figure 9A). The gene expression levels of *Col1a1*, *Col1a2*, *Col3a1,* and *Fn* were significantly increased, especially in homozygous female mice (Figure 9B–E). It is noteworthy that female mice showed increased levels of ECM, as SSc predominantly affects women. Since primary fibroblasts from female mice showed significantly increased expression of h*IGFBP-5*, *Col1a1*, *Col1a2*, and *Col3a1* as compared with primary fibroblasts from male mice, we examined the levels of the corresponding proteins in pulmonary fibroblasts of female mice. In primary lung fibroblasts, secreted hIGFBP-5 (hBP-5) was detected in culture supernatants, and levels in fibroblasts from homozygous mice were higher than those from heterozygous mice (Figure 9F,G). Levels of Col1α1 and Col3α1 paralleled mRNA levels with a notable increase in homozygous mice, and levels of FN significantly increased in both heterozygous and homozygous mice as compared with wild-type mice (Figure 9F,G), in contrast to *Fn* mRNA levels (Figure 9E). Levels of alpha-smooth muscle actin (α-SMA), a marker of fibroblast activation to myofibroblasts, showed a modest, but not significant, increase in fibroblasts from homozygous mice (Figure 9F). There was no noticeable difference in PCNA levels (Figure 9F), suggesting comparable proliferation of fibroblasts from the three different groups. This indicates that increased ECM production is not due to increased cell proliferation. Thus, transgene expression in cultured primary fibroblasts resulted in significant increases in ECM protein levels.

## 3. Discussion

Our group has contributed insights into increased IGFBP-5 levels in the setting of fibrosis and the role of IGFBP-5 in promoting a fibrotic phenotype. We previously showed that IGFBP-5 was overexpressed in dermal fibroblasts from patients with SSc and pulmonary fibroblasts from patients with IPF [7]. We also reported that IGFBP-5 levels were significantly increased in lung tissues and primary pulmonary fibroblasts of patients with SSc-associated pulmonary fibrosis [9]. We further demonstrated that IGFBP-5 could trigger a fibrotic phenotype in primary human pulmonary fibroblasts [7,8], in vivo in mouse skin and lung tissues [10,11], and ex vivo in human skin maintained in organ culture [5,12]. Our previous in vivo model relied on the use of adenovirally expressed human IGFBP-5. Therefore, we used the human IGFBP-5 sequence to generate transgenic mice, since mammalian IGFBP-5 is highly conserved between humans and mice.

To achieve a physiologically relevant expression pattern of h*IGFBP-5* in the transgenic mice, the transgene copy number was calculated. High and low copy numbers of the human transgene from both heterozygous and homozygous mice were used to characterize the transgenic mice. We anticipated that the expression patterns and the expression-dependent effects would likely provide a more extensive phenotype [21]. Our analysis showed that transgenic mice expressing higher levels of h*IGFBP-5* have increased gene expression levels in lung and skin tissues in both young and aged mice. Although the gene expression levels between lung and skin tissues were different, the overall effects of the transgene on ECM were similar, with consistent increases in *Col3a1*, *Fn,* and *Lox* levels. The histological analysis of the skin tissues showed increased dermal thickness in transgenic mice as compared with wild-type mice, with decreased hypodermal/adipose layer thickness. In addition, histological analysis of the lung tissues demonstrated increased cellularity and modest increases in ECM deposition. Further observation of the lung tissues showed mononuclear cell infiltration and ECM deposition in mice expressing high levels of the human transgene, suggesting that IGFBP-5 expression results in inflammation, an early phase in the fibrotic response. This observation was consistent with our previous report in which overexpression of IGFBP-5 in the mouse lung using an adenovirus resulted in mononuclear cell infiltration, fibroblast activation, and ECM deposition in vivo [11].

In comparison to the pronounced effects of adenovirally expressed IGFBP-5 in vitro using human primary lung fibroblasts [7,8] and ex vivo using human lung and skin tissues in fibrosis [5,9,12], the effects of transgenic expression of human IGFBP-5 in vivo were modest. However, when mice were grouped into those expressing high vs. low transgene levels, those expressing higher levels of human *IGFBP-5* had increased ECM levels and histological evidence of inflammation and fibrosis. One possible explanation is that the level of expression of the transgene in mice with low expression is insufficient to drive overt fibrosis and tissue remodeling, while it is enough to induce some increases in extracellular matrix gene expression. High expression of *IGFBP-5* resulted in increased ECM production in vivo and in vitro. This may be due to the fact that IGFBP-5 is expressed in diverse cell types, and its expression level is regulated by a variety of signaling pathways. Therefore, variable responses downstream of human IGFBP-5 in tissues could be due to different effects on different cell types. Furthermore, numerous proteases are reported to degrade IGFBP-5 including serine proteases [22], thrombin [22], complement 1s (C1s) [23], pregnancy-associated plasma protein-A (PAPP-A and PAPP-A2) [24,25], ADAM-9 and ADAM 12-S [26,27], mannan-binding lectin-associated serine protease 3 (MASP-3) [28], cathepsin G and elastase [29], matrix metalloproteases (MMP 1, 2 and 9) [30], plasmin [31], human kallikrein 2 and 3 (HK2 and 3) [32], and HtrA serine peptidase 1 (HTRA1) [33]. Thus, transgenic IGFBP-5 expression may not result in sufficient increases in the levels of corresponding protein due to degradation by proteases, and the proteolytic degradation is likely overcome in the mice expressing higher levels of the transgene. This may also explain why h*IGFBP-5* transgenic mice did not have an obvious overt phenotype as compared with adenoviral expression of the gene, which resulted in abundant mRNA and protein levels that could tip the balance in favor of intact protein.

Fibroblasts are essential effector cells responsible for the increased production of ECM, and thus fibrosis in different organs [34]. They have been widely used for assessing the effect of pro-fibrotic factors and for testing the efficacy of potential anti-fibrotic therapies [9,34]. As we previously reported [7], overexpression of IGFBP-5 in normal primary fibroblasts or treatment with recombinant IGFBP-5 increased ECM deposition. To further characterize the phenotype of transgenic h*IGFBP-5* mice, we employed a complementary approach by using primary lung fibroblasts cultured from lung tissues of transgenic h*IGFBP-5* mice. In primary lung fibroblasts, we saw a more consistent increase in ECM genes that was more comparable to the effects noted with recombinant and adenoviral IGFBP-5 [7]. This further suggests that fibroblasts can respond to IGFBP-5 by increasing ECM production, and the in vitro response is likely due to reduced IGFBP-5 degradation by proteases secreted by other cell types in vivo. This also suggests that higher h*IGFBP-5* transgene expression levels are required to sufficiently induce an overt fibrotic phenotype. In fact, data from our lung and skin tissues showed that mice that had higher transgene expression also showed increased expression levels of collagen, fibronectin, and LOX, suggesting that higher IGFBP-5 levels correlated with elevated ECM production and a fibrotic phenotype. A comprehensive high throughput analysis of genes regulated by IGFBP-5 in fibroblasts from the transgenic mice would provide additional insights on genes, pathways, and networks regulated by the transgene.

In our study, the levels of endogenous mouse *Igfbp-5* were variable among the groups. It is not known whether the human transgene levels affect endogenous mouse *Igfbp-5* expression. However, we previously showed that IGFBP-5 could increase its own expression to generate a feedback loop, and it could act in concert with other growth factors, such as connective tissue growth factor (CTGF), to drive fibrosis and tissue remodeling [9]. Therefore, it is plausible for the human transgene to regulate the expression of endogenous mouse *Igfbp-5,* since levels of the endogenous gene showed an increasing trend in the transgenic mice. In bleomycin-induced pulmonary fibrosis, in our study model, expression of endogenous mouse *Igfbp-5* was downregulated. One explanation is that bleomycin reduced endogenous mouse *Igfbp-5* levels as a negative feedback mechanism, although further investigation is warranted. Our data also showed a decrease in transgene expression by bleomycin. This suggests that bleomycin negatively regulated *EF1α* expression as the *EF1α* promoter was used to drive transgene expression.

Our study is the first to report ubiquitous transgenic expression of human *IGFBP-5* and describe the phenotype of the transgenic mice. Since the effects of IGFBP-5 are known to be tissue specific, targeted transgenic expression of *IGFBP-5* in specific tissues such as bone and mammary gland has been reported [35]. However, transgenic mice overexpressing IGFBP-5 in specific tissues exhibited a less severe phenotype [35]. Transgenic mice overexpressing IGFBP-5 under the control of the osteocalcin promoter (OC-rIGFBP-5) targeting it to bone had a transient decrease in trabecular bone volume secondary to reduced trabecular number and thickness and a transient decrease in bone mineral apposition rate [36]. Furthermore, as compared with older transgenic mice, younger OC-rIGFBP-5 transgenic mice showed a reduction of total, vertebral, and femoral bone mineral densities. These changes correlated with decreased expression of bone markers [36]. Transgenic mice overexpressing IGFBP-5 in the mammary gland using a mammary specific promoter, β-lactoglobulin, showed impaired mammary gland development and function [37]. This study was the first to demonstrate the important role of IGFBP-5 in mammary development and to show evidence that IGFBP-5 inhibits cellular proliferation and induces cell death when expressed in the mammary glands of mice [37].

Our employment of the transgenic model was used to study fibrosis. Lung fibrosis such as IPF is more prevalent in aging populations, with a sharp increase in incidence in those older than 50 [38]. IPF shares common pathophysiologic mechanisms with the aging process including genomic instability, telomere attrition, epigenetic alterations, loss of proteostasis, deregulated nutrient sensing, mitochondrial dysfunction, cellular senescence, and stem cell exhaustion [39]. Cellular senescence is reportedly higher in lung tissues of older mice than young mice at baseline and increases in experimental lung injury and fibrosis [40]. A study by Redente et al. suggested that the variables of advanced age and male sex contributed to the severity of pulmonary fibrosis [18]. Their study showed that aged male mice developed more severe lung disease, with increased mortality, increased collagen deposition, and neutrophilic alveolitis as compared with aged female mice or young mice of either sex [18]. Schaferet al. also reported that fibrotic lung disease was mediated in part by senescent cells [41]. The role of cellular senescence in fibrosis is complicated, particularly as cellular senescence is protective in certain situations. For example, cellular senescence has a tumor suppressor property in cancer and has also been suggested to suppress fibrosis [42]. To support this claim, a study by Krizhanovsky et al. showed cellular senescence acted to limit the fibrogenic response to tissue damage [43]. According to the study, upon liver injury, senescent cells derived from hepatic stellate cells proliferated and secreted ECM components resulting in a fibrotic scar that eventually resolved [43]. This senescent response exhibited patterns of decreased ECM production and increased secretion of ECM-degrading matrix metalloproteases [43]. These findings suggest that cellular senescence could be considered to be a beneficial compensatory response to the damage caused by fibrosis and aging, especially when tissues exhaust their regenerative capacity [40]. These factors could explain, at least in part, why our “aged” mice did not have an overt fibrotic phenotype. The ECM production could be declining over time, whereas the MMP and other protease secretion increase, especially since several proteases, including some MMPs, are reported to degrade IGFBP-5.

The fact that male sex exacerbates fibrosis is also supported by our findings where male mice had thicker skin as compared with female mice, irrespective of genotype. This may, in part, explain the male predominance in IPF [44] and the fact that SSc can be more severe in men even though it is more prevalent in women [45].

Genetic ablation of IGFBP-5 has yielded limited phenotypes, and this could be due to compensation by remaining members of the IGFBP family [46]. For example, we have shown that both IGFBP-3 and IGFBP-5 could induce fibrosis [7] and loss of one these proteins in vivo may be compensated for by the other.

In summary, mice with ubiquitous transgenic expression of human *IGFBP-5* exhibited increases in ECM gene expression. These findings suggest elevation of extracellular matrix protein production could be one of the early stages in the development of fibrosis, and additional insults may be needed to obtain an overt fibrotic phenotype. Our findings also suggest that higher levels of *IGFBP-5* are needed to induce a fibrotic phenotype, likely as a result of the increasing number of proteases that degrade the IGFBP-5 protein. We hope that the availability of these mice will facilitate additional research on the effects of IGFBP-5 in vivo.

## 4. Materials and Methods

### 4.1. Ethics Statement

All of the animal experiments were approved by the Medical University of South Carolina (MUSC) Institutional Animal Care and Use Committee (IACUC).

### 4.2. Generation of Transgenic Mice Overexpressing Human IGFBP-5

Human cDNA encoding *IGFBP-5* was cloned, as previously described [5] and the sequence was confirmed. The construct was used by Cyagen to generate the transgenic mice (Cyagen Bioscience, Santa Ana, CA, USA). In brief, the *Rosa26* locus in the mouse genome was used as target site to co-inject donor DNA containing human *IGFBP-5* cDNA and *Cas9* mRNA into fertilized mouse eggs to generate targeted knock-in offspring. F_0_ founder animals were identified by PCR of tail DNA followed by sequence analysis and were bred to wild-type mice to test germ-line transmission and F_1_ animal generation. The gene targeting in F_0_ animals was further confirmed by Southern blot analysis. The mice were bred according to established protocols. Different pairs of male and female mice were placed in a single cage for mating. Litters from each pair were weaned after 21 days and their tail biopsy DNA was used for genotyping. The animals were backcrossed for over 10 generations onto the C57BL/6J genetic background.

### 4.3. Mouse Strains and Husbandry

Mice were singly housed in a temperature- and humidity-controlled barrier facility. The mice were maintained on a 12-h reverse dark/light cycle. Animals had free access to food and water.

### 4.4. Mouse Model for Oral Bleomycin (BLM) Delivery

Male and female mice that were 8 weeks old were given pharmaceutical-grade bleomycin (BLM) (Hospira Inc., Lake Forest, IL, USA) via the oropharyngeal route. Phosphate-buffered saline (PBS) or BLM dissolved in PBS at concentrations of 1.5 U/kg were administered. Mouse weight was monitored every 3 days. Mice were sacrificed on day 14 after BLM administration. Lung and skin tissues were harvested for histological and gene expression analyses.

### 4.5. Mouse Specimen Collection

Mice were euthanized by the inhalation of CO_2_ method followed by cervical dislocation to ensure euthanasia. All tissues were examined grossly and collected. Heart, lung, kidneys, and liver were weighed. Skin and lung tissues were harvested and frozen for gene expression or fixed in 10% formalin and embedded in paraffin for histology. All remaining organs were immersion-fixed in 10% neutral buffered formalin and embedded in paraffin for histology. Harvested lung tissues were inflated in 10% formalin prior to paraffin-embedding.

### 4.6. Identification of Mouse Genotype

#### 4.6.1. Genomic DNA Isolation from Mouse Tail Biopsy

A small mouse tail biopsy (2–3 mm) was collected and genomic DNA was isolated. Briefly, each tissue piece was boiled in 600 µL of 50 mM NaOH for 10 min at 100 °C. Samples were vortexed for 10 s and 100 µL of 1 M Tris-Cl, pH 6.8 was added to bring the pH to about 8. The solution was mixed and centrifuged at 12,000 rpm for 6 min at room temperature to pellet debris. Supernatant was carefully transferred to a fresh tube and stored at −80 °C or used for genotyping using PCR.

#### 4.6.2. Polymerase Chain Reaction

The PCR reaction consisted of genomic DNA from mouse tail biopsy, forward and reverse primers (Integrated DNA Technologies, Coralville, IA, USA), as listed in Table 1, 10× DreamTaq Green Buffer and DreamTaq DNA Polymerase (ThermoFisher Scientific, Waltham, MA, USA), 10 mM dNTP (Invitrogen, Carlsbad, CA, USA), and distilled H_2_O. Samples were denatured at 94 °C for 30 s, annealed at 60 °C for 30 s, and extended at 65 °C for 45 s for 40 cycles. PCR products were visualized on a 1% agarose gel.

### 4.7. Histological Analysis

After dissection, all tissues were fixed in 10% formalin and embedded in paraffin. Six micrometer sections of paraffin-embedded mouse skin and lung specimens were placed on glass slides and stained with hematoxylin and eosin (H&E). A subset of lung and skin sections were stained with Masson’s trichrome. H&E and Masson’s trichrome-stained sections of tissues were analyzed by a board-certified veterinary pathologist. Images were captured on an Axio Observer Microscope (Carl Zeiss Microscopy GmbH, Oberkochen, Germany) using identical camera setting.

### 4.8. Culture of Primary Mouse Lung Fibroblasts

Primary mouse fibroblasts from lung tissues of mice were cultured in Dulbecco’s modified Eagle’s medium (DMEM) (Mediatech, Inc., Manassas, VA, USA) supplemented with 10% fetal bovine serum (FBS) (Sigma-Aldrich, St. Louis, MO, USA), penicillin, streptomycin, and antimycotic agent (Invitrogen, Carlsbad, CA, USA), as previously described [20]. Briefly, the lung tissues were minced, and pieces were allowed to adhere to 100 mm tissue culture dishes for one hour prior to the addition of culture medium. The lung tissues were cultured at 37 °C in a humidified atmosphere. Fibroblasts migrating out of the tissues were allowed to populate the culture dish. Once confluent, the fibroblasts were passaged, into a T-175 culture flask. For experiments, 2.0 × 10^5^ primary fibroblasts were plated in 6-well tissue culture plates in 10% FBS-containing DMEM. After 96 h, supernatants and cellular lysates were harvested. Cells were used in passages 3 to 7.

### 4.9. Quantitative Real-Time RT-PCR

Total RNA was extracted from mouse lung and skin tissues using a RNeasy^®^ mini kit (Qiagen Inc., Valencia, CA, USA). First-strand cDNA was reverse-transcribed with an oligo (dT)12-15 primer (Invitrogen, Carlsbad, CA, USA) and SuperScript IV Reverse Transcriptase (Invitrogen). Gene mRNA expression levels were evaluated by quantitative PCR using the TaqMan^®^ real-time PCR system (Applied Biosystems, Foster City, CA, USA), according to the manufacturer’s protocol. Gene expression levels were normalized to beta-2-microglobulin (*B2m*) (Mm00437762_m1). Relative expression levels were compared using the comparative CT method formula 2−ΔΔCt. Specific primers and probes for amplifying genes encoding human *IGFBP-5* (Hs00181213_m1); mouse *Lox* (Mm00495386_m1); mouse *Igfbp-5* (Mm00516037_m1); mouse collagen1a1, *Col1a1* (Mm00801666_g1); mouse collagen 1a2, *Col1a2* (Mm00483888_m1); mouse collagen 3a1, *Col3a1* (Mm00802300_m1); mouse fibronectin, *Fn* (Mm01256744_m1); and mouse interleulin-6, *Il-6* (Mm00446190_m1) were used. Mouse *Gapdh* (Mm99999915_g1) was also used to confirm results obtained with *B2m* with no notable differences (data not shown).

### 4.10. Western Blot

Conditioned media and cell lysates from cultured primary lung fibroblasts were analyzed by immunoblotting. The following antibodies were used: fibronectin (FN) monoclonal antibody (clone EP5) (Santa Cruz, Dallas, TX, USA), collagen alpha (I) (Col1α1) polyclonal antibody (Abnova, Taipei City, Taiwan), collagen alpha (III) (Col3α1) polyclonal antibody (Novus Biologicals, Centennial, CO, USA), human IGFBP-5 polyclonal antibody (GroPep Bioreagents, Thebarton, SA, Australia), alpha smooth muscle actin (α-SMA) polyclonal antibody (Abcam, Cambridge, UK), proliferating cell nuclear antigen (PCNA) (Abcam, Clone ab29), and GAPDH monoclonal antibody (Santa Cruz) as primary antibodies and horseradish peroxidase-conjugated antibody as a secondary antibody. Signals were detected using chemiluminescence on FluorChem R System (ProteinSimple, San Jose, CA, USA). Densitometry was analyzed with ImageJ software (U.S. National Institutes of Health, Bethesda, MD, USA).

### 4.11. Statistical Analysis

All continuous variables were expressed as the mean ± standard deviation. All statistical analyses were done using GraphPad Prism version 8 for Windows (GraphPad Software, La Jolla, CA, USA). As appropriately indicated in the figure legends, comparison among 3 or more groups was performed using ANOVA followed by Tukey’s multiple comparisons test for Figure 3, Figure 4, and Figure 6. Due to undetected values of wild-type in Figure 2, Figure 6A, Figure 8A and Figure 9A, data were analyzed using unpaired *t*-test for comparison between the hIGFBP-5 levels in the heterozygous and homozygous groups. Comparisons among 3 groups with additional factors such as treatments (PBS and BLM) or genders (male and female) were performed using two-way ANOVA followed by Tukey’s multiple comparisons test for Figure 8 and Figure 9. *p*-values ≤ 0.05 were considered to be statistically significant.

## Figures and Tables

**Table 1:** Genotyping primers.

Forward Primer 1	5′-AAGCACGTTTCCGACTTGAGTTG-3′
Reverse Primer 1	5′-GGGTGAGCATGTCTTTAATCTACC-3′
Reverse Primer 2	5′-GAGCCAGTACACGACATCACTTTC-3′

## Data Availability

Data is contained within the article and supplementary material.

## Supplementary Materials

Supplementary materials can be found at https://www.mdpi.com/1422-0067/22/1/335/s1.

## Abbreviations

α-SMA	Alpha-smooth muscle actin
B2m	Beta-2-microglobulin
BLM	Bleomycin
Col1a1	Collagen type 1α1
Col1a2	Collagen type 1α2
Col3a1	Collagen type 3α1
CTGF	Connective tissue growth factor
ECM	Extracellular matrix
EF1α	Elongation factor 1 alpha
Fn	Fibronectin
H&E	Hematoxylin and eosin
HET	Heterozygous
HOMO	Homozygous
hIGFBP-5	Human IGFBP-5
IGF-1, 2	Insulin growth factor-1, 2
IGFBP-5	Insulin-like factor binding protein-5
IPF	Idiopathic pulmonary fibrosis
Il-6	Interleukin-6
Lox	Lysyl oxidase
mIgfbp-5	Mouse Igfbp-5
PBS	Phosphate-buffered saline
PCNA	Proliferating cell nuclear antigen
SSc	Systemic sclerosis
WT	Wild-type
