# Supplementary material for: Phenotypic Characterization of Transgenic Mice Expressing Human IGFBP-5
Source: Int J Mol Sci. 2020 Dec 30;22(1):335. doi: 10.3390/ijms22010335 (PMC7795366; doi:10.3390/ijms22010335)
Supplement: Supplementary file 1 [file ijms-22-00335-s001.pdf]

Appendix A

Supplemental Figures

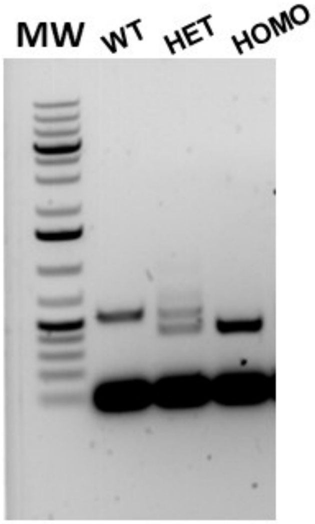

Supplemental Figure 1: Genotype of transgenic mice on agarose gel. PCR products of genomic DNA extracted from tail biopsy of transgenic mice. The samples were visualized on an agarose gel. The key information is noted as: MW- DNA ladder, WT- wild-type, HET- heterozygous, HOMO- homozygous. The image shows the full length of the gel corresponding to the cropped image in figure 1.

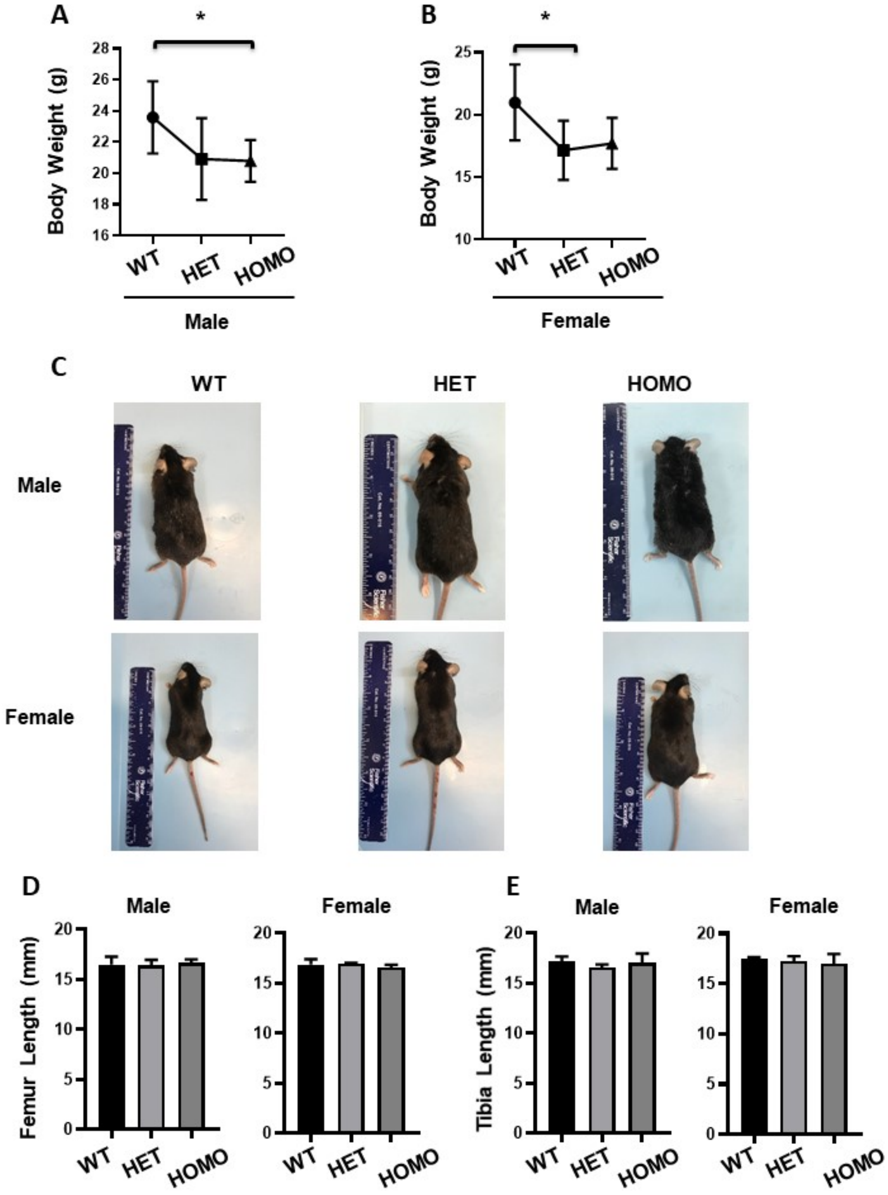

Supplemental Figure 2: Generation and characterization of transgenic mice. (A) Body weight of male mice at 8 weeks. Data obtained from 5 WT, 7 HET, 7 HOMO mice. Comparison among 3 or more groups was performed using ANOVA followed by Turkey's multiple comparison test. Values represent mean ± standard deviation. *P < 0.05. (B) Body weight of female mice at 8 weeks. N=7 per group. Comparison among 3 or more groups was performed using ANOVA followed by Turkey's multiple comparison test. Values represent mean ± standard deviation. *P < 0.05. (C) Whole body picture of both male and female mice. (D) Measurement of right femur length in both male and female mice. N = 3 per group. Comparison among 3 or more groups was performed using ANOVA followed by Turkey's multiple comparison test. Values represent mean ± standard deviation. No significant differences were noted. (E) Measurement of right tibia length in both male and female mice. N = 3 per group. Comparison among 3 or more groups was performed using ANOVA followed by Turkey's multiple comparison test. Values represent mean ± standard deviation. No significant differences were noted.

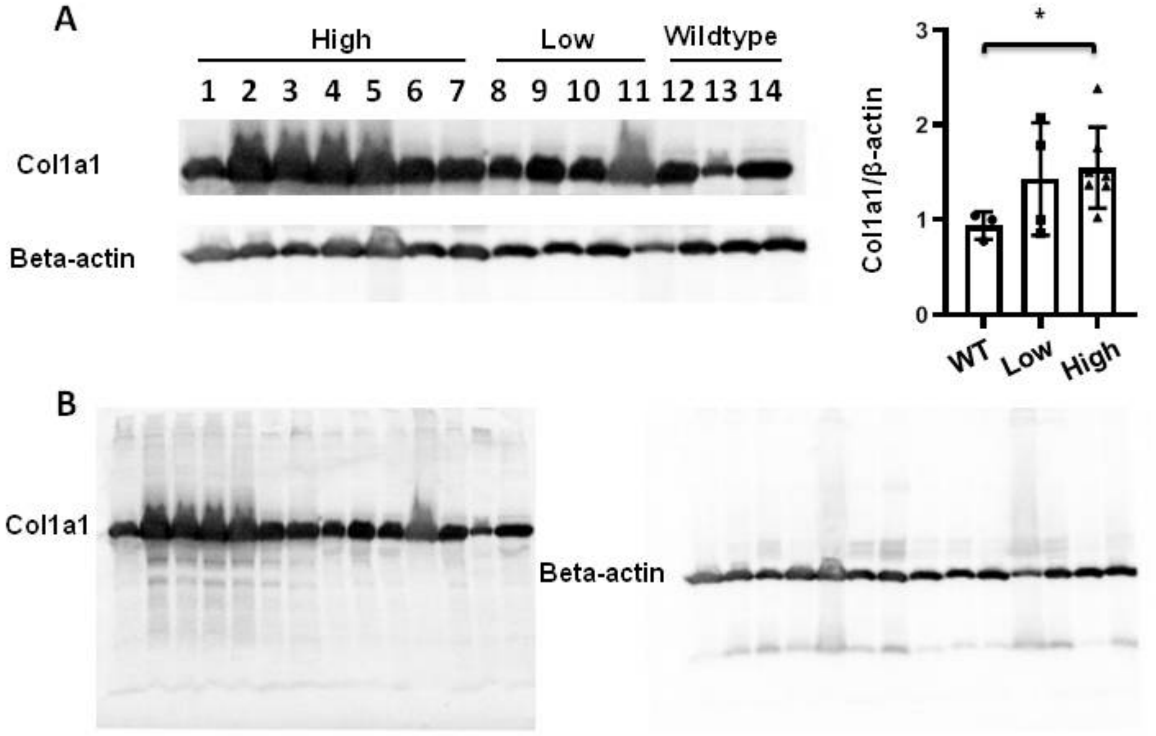

Supplemental Figure 3: Increased Col1a1 protein levels in high transgene-expressing mice. Lung homogenates were prepared from mice expressing high and low levels of *hIGFBP-5* and wild-type mice. (A) Immunoblots of lung homogenates were probed with Col1a1 antibody and normalized to beta-actin antibody. The data were obtained from lung homogenates of 3 WT, 4 low *hIGFBP-5*-expressing and 7 high *hIGFBP-5*-expressing mice. Statistical comparison was performed using one-way ANOVA followed by Tukey's multiple comparisons test. Values represent mean ± standard deviation. *$P < 0.05$. (B) Full images of Collagen1a1 and beta-actin blots shown in A.

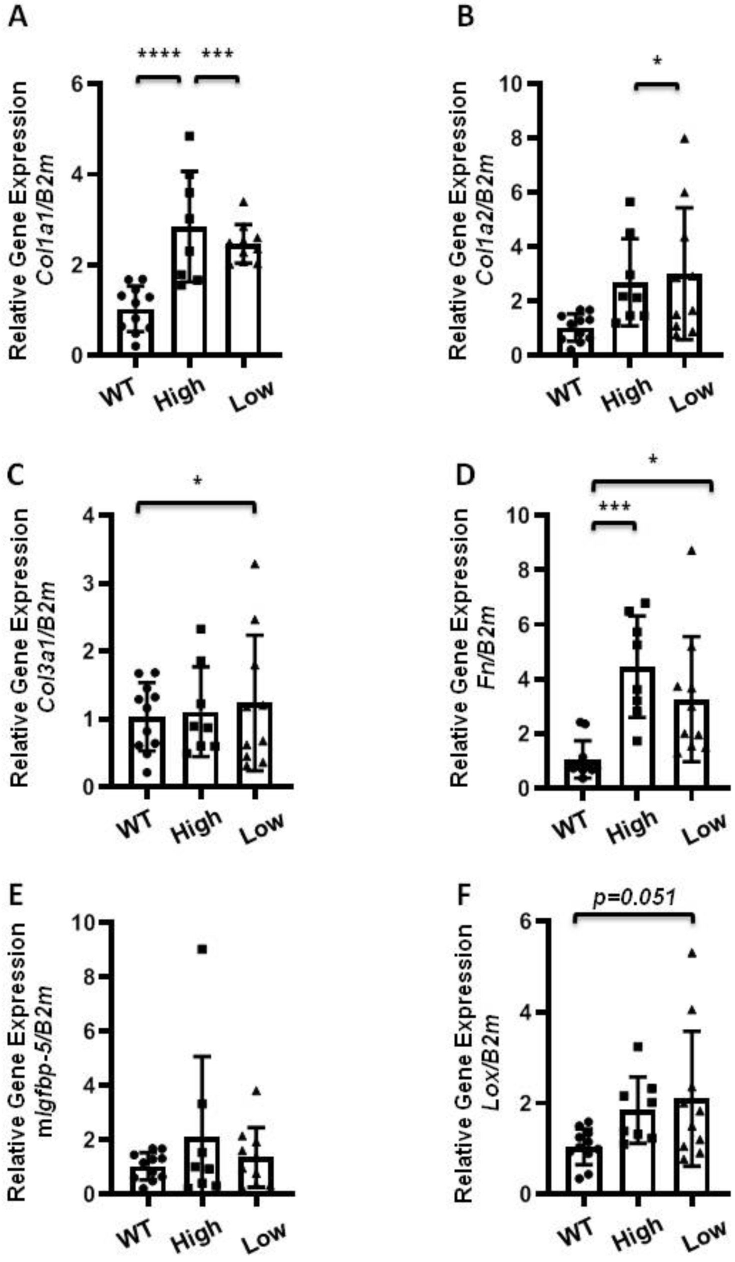

Supplemental Figure 4: ECM gene expression in lung tissues of heterozygous mice. Lung tissues of heterozygous male and female mice at 8 weeks of age were harvested to measure baseline expression using qPCR. The following genes were analyzed: (A) *Col1a1*. (B) *Col1a2*. (C) *Col3a1*. (D) *Fn*. (E) mouse *Igfbp-5*. (F) *Lox*. Data obtained from 11 WT (6 M and 5 F), 8 High h*IGFBP-5*-expressing and 10 Low h*IGFBP-5*-expressing mice. Statistical comparison was performed using one-way ANOVA followed by Tukey's multiple comparisons test. Values represent mean ± standard deviation. *P < 0.05. **P < 0.01. ***P < 0.001. ****P < 0.0001.

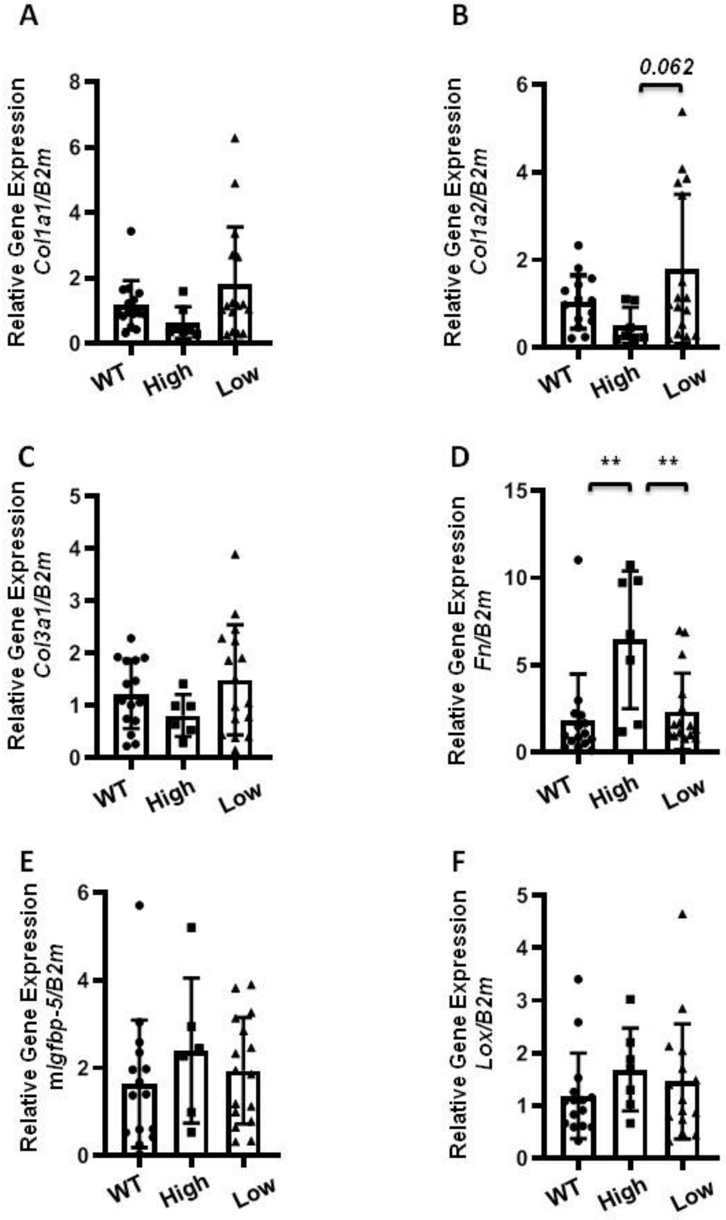

Supplemental Figure 5: ECM gene expression in skin tissues of heterozygous mice. Skin tissues of 8-week-old heterozygous male and female mice were harvested to measure baseline expression using qPCR. The following genes were analyzed: (A) *Col1a1*. (B) *Col1a2*. (C) *Col3a1*. (D) *Fn*. (E) mouse *Igfbp-5*. (F) *Lox*. Data obtained from 15 WT (8 M and 7 F), 7 High h*IGFBP-5*-expressing and 16 Low h*IGFBP-5*-expressing mice. Statistical comparison was performed using one-way ANOVA followed by Tukey's multiple comparisons test. Values represent mean ± standard deviation. **P < 0.01.

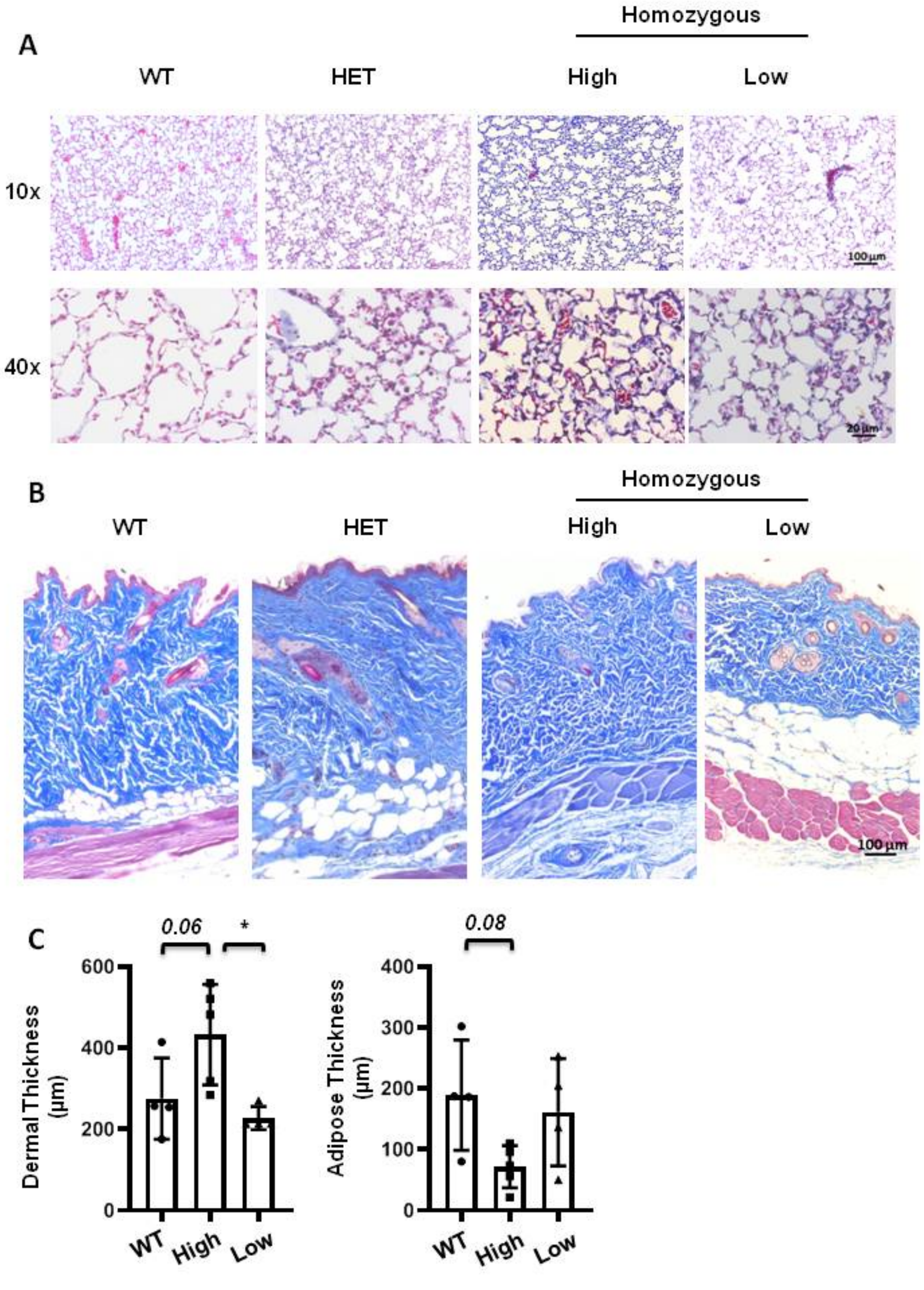

Supplemental Figure 6: Masson Trichrome staining in lung and skin tissues of 8-week-old mice. (A) Lung tissues of wild-type, heterozygous and homozygous mice expressing high and low levels of *hIGFBP-5*. Magnification: 10x, Scale bar: 100 µm, and Magnification: 40x, Scale Bar: 20 µm. (B) Skin tissues of wild-type, heterozygous and homozygous mice expressing high and low levels of *hIGFBP-5*. Magnification, 10x. Scale bar, 100 µm. (C) Dermal and adipose thickness were measured in 4 WT, and 5 high *hIGFBP-5*-expressing and 4 low *hIGFBP-5*-expressing mice. Statistical comparison was performed using one-way ANOVA followed by Tukey's multiple comparisons test. Values represent mean ± standard deviation. *P < 0.05.

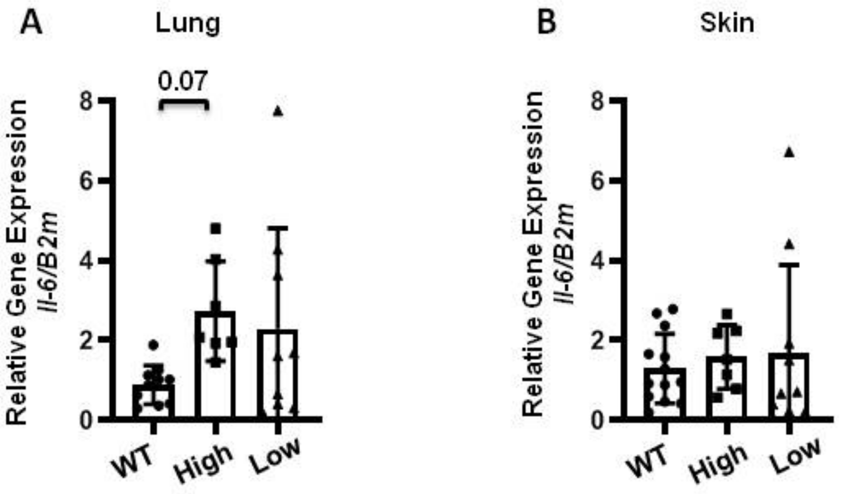

Supplemental Figure 7: Increased *Il-6* expression in lung tissues of mice expressing high levels of *hIGFBP-5*. (A) Lung tissues of 8-week-old wild-type and homozygous mice expressing high and low levels of *hIGFBP-5* were harvested to measure baseline expression using qPCR. Data obtained from 11 WT (6 M and 5 F), 7 High *hIGFBP-5* expressing and 9 Low *hIGFBP-5* expressing mice. Statistical comparison was performed using one-way ANOVA followed by Tukey's multiple comparisons test. Values represent mean ± standard deviation. *$P < 0.05$. (B) Skin tissues of 8-week-old wild-type and homozygous mice expressing high and low levels of *hIGFBP-5* were harvested to measure baseline expression using qPCR. Data obtained from 15 WT (8 M and 7 F), 7 High *hIGFBP-5*-expressing and 11 Low *hIGFBP-5*-expressing mice. Statistical comparison was performed using one-way ANOVA followed by Tukey's multiple comparisons test. Values represent mean ± standard deviation. No significant difference was noted.

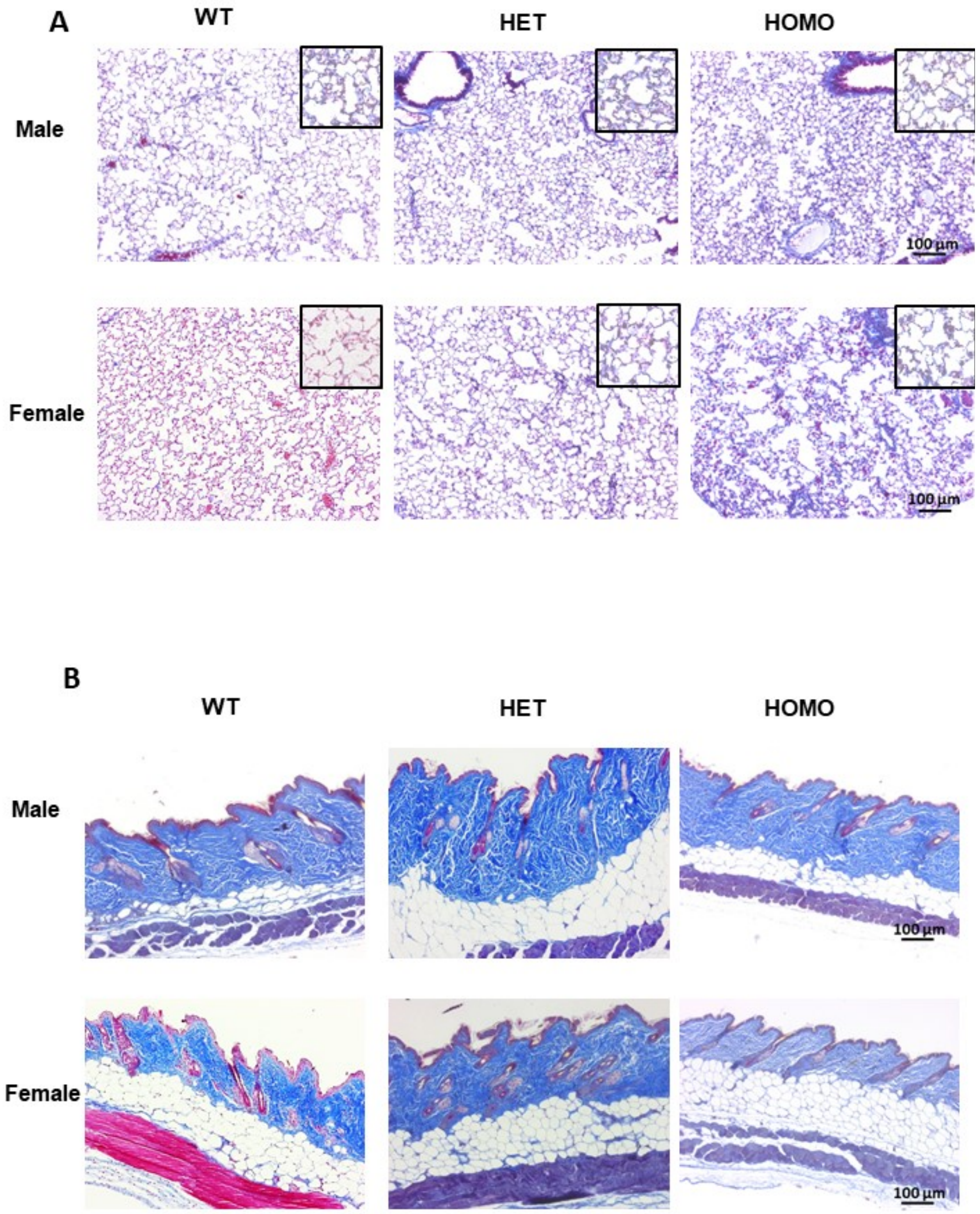

Supplemental Figure 8: Masson Trichrome staining in lung and skin tissues of aged mice. (A) Lung tissues. (B) Skin tissues. Magnification, 10x. Scale bar, 100 µm, and Magnification: 40x, Scale bar: 20 µm.

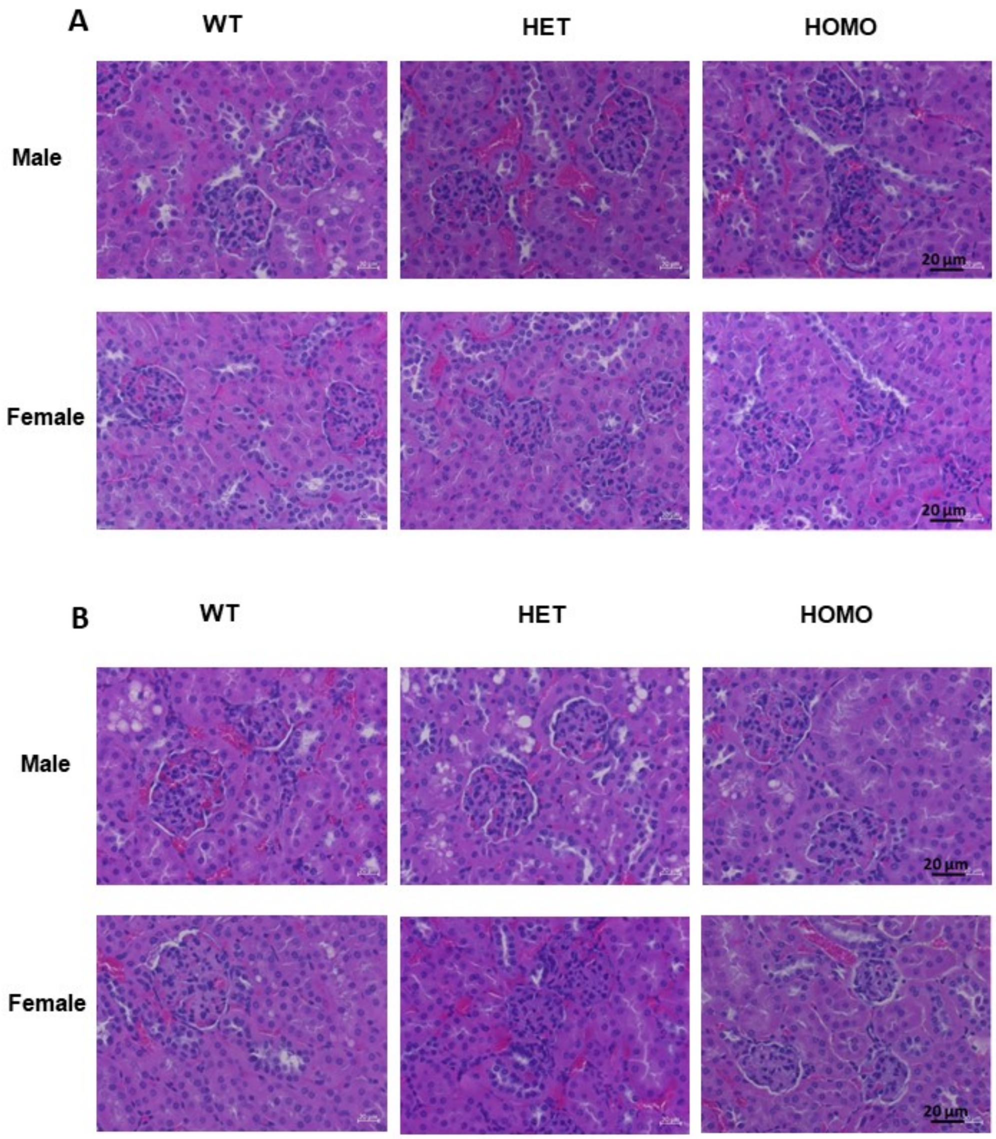

Supplemental Figure 9: Histological evaluation of tissue morphology in kidneys from 10-week-old and 1-year-old transgenic mice. (A) Representative images of kidney tissues from 10-week-old wild-type and transgenic mice from both sexes. (B) Representative images of kidney tissues from 1-year-old wild-type and transgenic mice from both sexes. Magnification 40x. Scale bar, 20 µm.

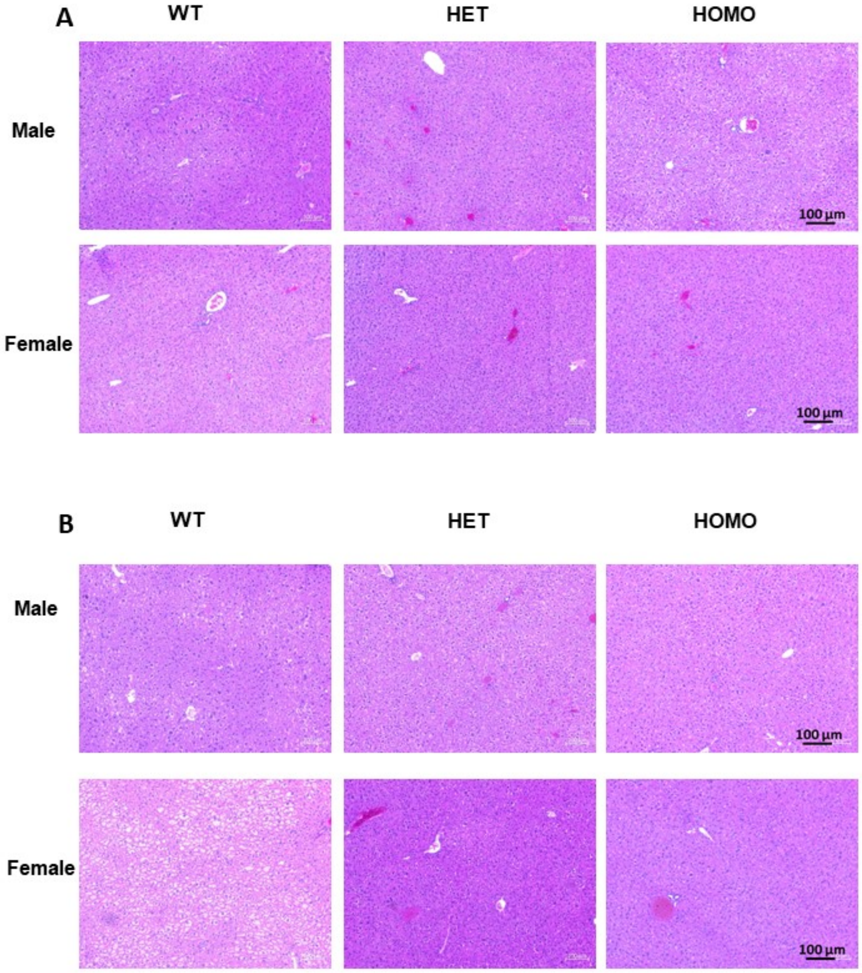

Supplemental Figure 10: Histological evaluation of tissue morphology in livers from 10-week-old and 1-year-old transgenic mice. (A) Representative images of liver tissues from 10-week-old wild-type and transgenic mice from both sexes. (B) Representative images of liver tissues from 1-year-old wild-type and transgenic mice from both sexes. Magnification 10x. Scale bar, 100 µm.

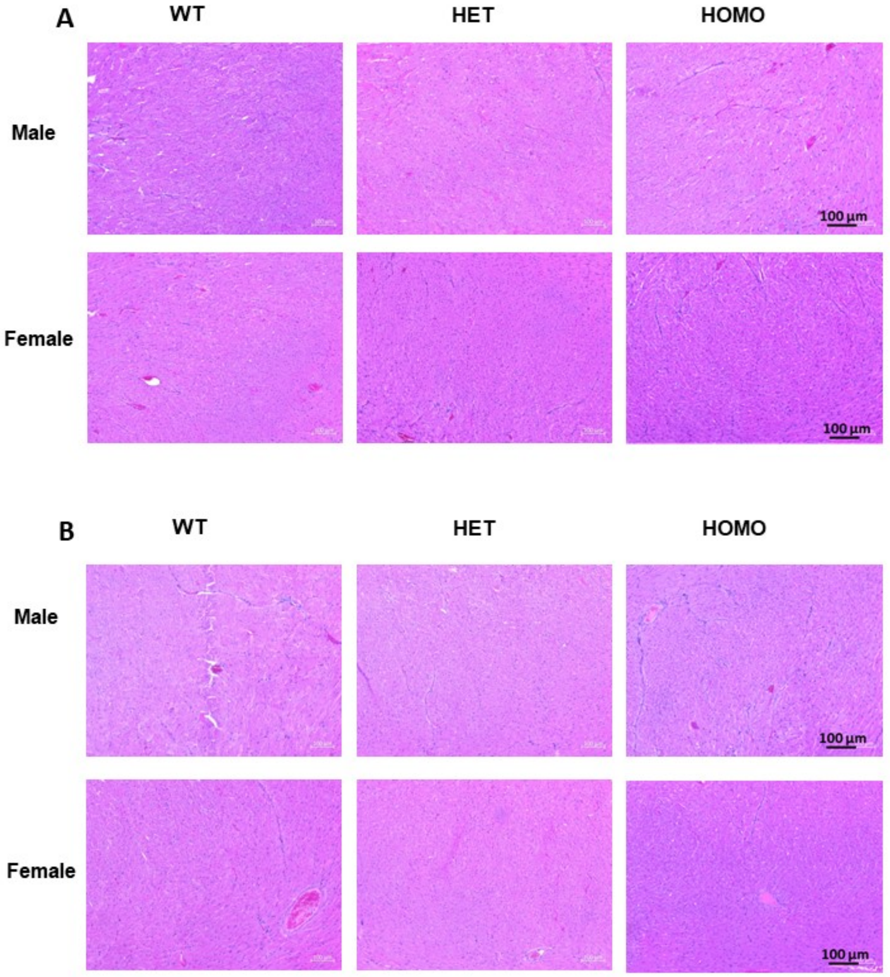

Supplemental Figure 11: Histological evaluation of tissue morphology in hearts from 10-week-old and 1-year- transgenic mice. (A) Representative images of heart tissues from a 10-week-old wild-type mice and transgenic mice from both sexes. (B) Representative images of heart tissues from a 1-year-old wild-type and transgenic mice from both sexes. Magnification 10x. Scale bar, 100 μm.

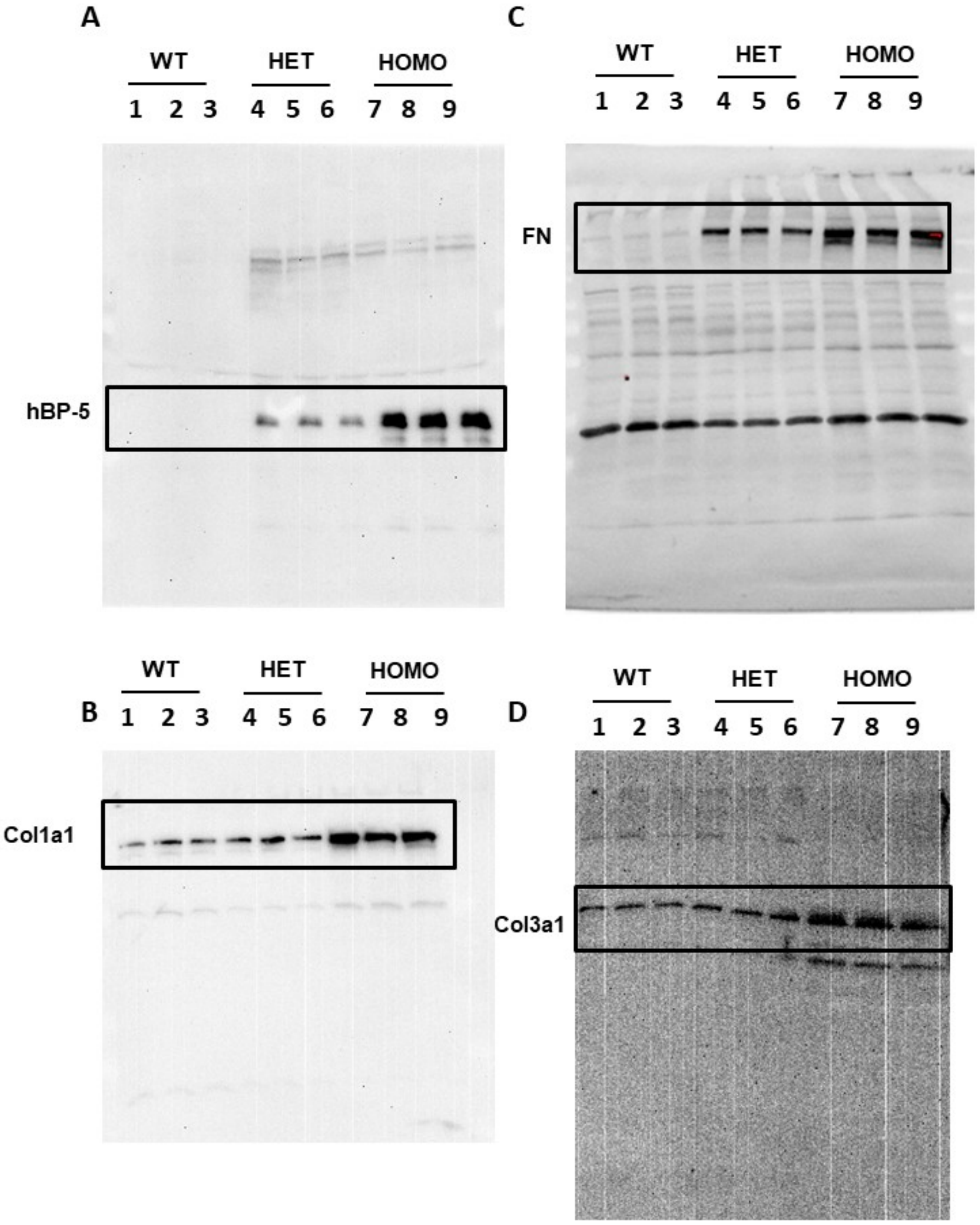

Supplemental Figure 12: Full uncropped images of western blots shown in Figure 9.

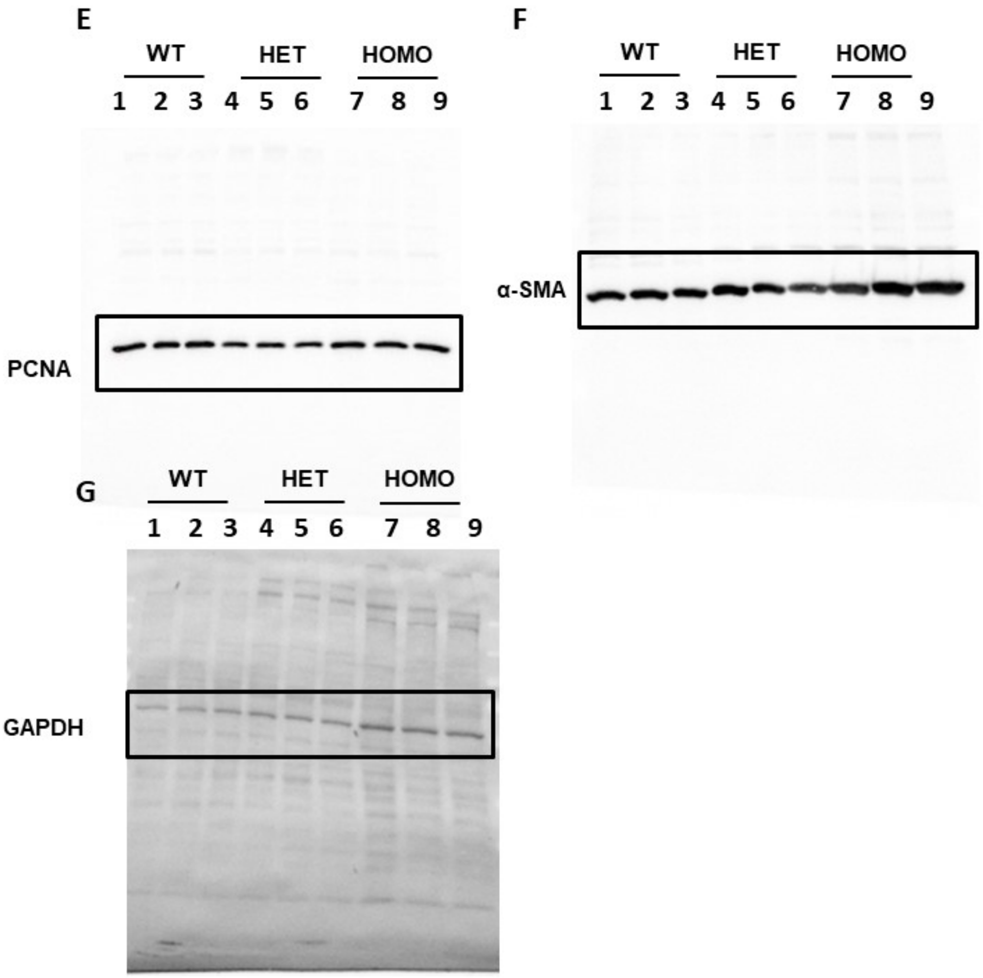

Supplemental Figure 13: Full uncropped images of western blots shown in Figure 9.

Appendix B

Supplemental Methods

B.1. Physical Examinations and Necropsy of Transgenic Mice

Wild-type and transgenic mice from both sexes at 10 weeks old and one-year old were used for necropsy. Mice were weighed prior to necropsy. Mice were euthanized by inhalation of CO_2 method followed by cervical dislocation to ensure euthanasia. Each organ was weighed and examined for irregularity. The tissues were fixed in 10% formalin and embedded in paraffin. **Six micrometer** sections of paraffin-embedded mouse kidney, liver and heart specimens were stained with hematoxylin and eosin (H&E) and Masson's Trichrome. Images were captured on an Axio Observer Microscope (Carl Zeiss Microscopy GmbH, Germany) using identical camera settings.

B.2. Western Blot

Lung tissues from wild-type and homozygous mice expressing low and high levels of *hIGFBP-5* were homogenized and analyzed by immunoblotting. The following antibodies were used: collagen type I (Col) polyclonal antibody (Cedarlane, Ontario, Canada), and beta-actin monoclonal antibody (Santa Cruz) as primary antibodies and horseradish peroxidase-conjugated antibody as a secondary antibody. Signals were detected using chemiluminescence on FluorChem R System (ProteinSimple, San Jose, CA, USA). Densitometry was analyzed with ImageJ software (U.S. National Institutes of Health, Bethesda, MD, USA).
